# PML nuclear body-residing proteins sequentially associate with HPV genome after infectious nuclear delivery

**DOI:** 10.1371/journal.ppat.1007590

**Published:** 2019-02-25

**Authors:** Lucile Guion, Malgorzata Bienkowska-Haba, Stephen DiGiuseppe, Luise Florin, Martin Sapp

**Affiliations:** 1 Department of Microbiology and Immunology, Center for Molecular and Tumor Virology, Feist-Weiller Cancer Center, Louisiana State University Health Sciences Center, Shreveport, Louisiana, United States of America; 2 Department of Microbiology-Immunology, Feinberg School of Medicine, Northwestern University, Chicago, Illinois, United States of America; 3 Department of Virology and Research Center for Immunotherapy (FZI), University Medical Center of the Johannes Gutenberg University Mainz, Mainz, Germany; University of Wisconsin-Madison, UNITED STATES

## Abstract

Subnuclear promyelocytic leukemia (PML) nuclear bodies (NBs) are targeted by many DNA viruses after nuclear delivery. PML protein is essential for formation of PML NBs. Sp100 and Small Ubiquitin-Like Modifier (SUMO) are also permanently residing within PML NBs. Often, large DNA viruses disassemble and reorganize PML NBs to counteract their intrinsic antiviral activity and support establishment of infection. However, human papillomavirus (HPV) requires PML protein to retain incoming viral DNA in the nucleus for subsequent efficient transcription. In contrast, Sp100 was identified as a restriction factor for HPV. These findings suggested that PML NBs are important regulators of early stages of the HPV life cycle. Nuclear delivery of incoming HPV DNA requires mitosis. Viral particles are retained within membrane-bound transport vesicles throughout mitosis. The viral genome is released from transport vesicles by an unknown mechanism several hours after nuclear envelope reformation. The minor capsid protein L2 mediates intracellular transport by becoming transmembranous in the endocytic compartment. Herein, we tested our hypothesis that PML protein is recruited to incoming viral genome prior to egress from transport vesicles. High-resolution microscopy revealed that PML protein, SUMO-1, and Sp100 are recruited to incoming viral genomes, rather than viral genomes being targeted to preformed PML NBs. Differential immunofluorescent staining suggested that PML protein and SUMO-1 associated with transport vesicles containing viral particles prior to egress, implying that recruitment is likely mediated by L2 protein. In contrast, Sp100 recruitment to HPV-harboring PML NBs occurred after release of viral genomes from transport vesicles. The delayed recruitment of Sp100 is specific for HPV-associated PML NBs. These data suggest that the virus continuously resides within a protective environment until the transport vesicle breaks down in late G1 phase and imply that HPV might modulate PML NB assembly to achieve establishment of infection and the shift to viral maintenance.

## Author summary

Promyelocytic leukemia (PML) nuclear bodies (NBs) are often targeted and reorganized by DNA viruses to counteract their antiviral activity. Human papillomavirus (HPV) also associates with PML NBs after infectious entry. While PML protein is required for nuclear retention and efficient transcription of incoming HPV genomes, Sp100, another PML NB component, was identified as a restriction factor. HPV virions are delivered to the nucleus during mitosis while continuously residing in membrane-bound transport vesicles. L2 protein directs trafficking via its carboxyl terminus by becoming transmembranous in the endocytic compartment. Herein, we demonstrate that PML protein associates with viral particles still residing in transport vesicles after nuclear delivery, possibly to provide a continuous protective environment after disruption of the membrane bilayer of the transport vesicle. In contrast, Sp100 recruitment is delayed specifically for PML NBs forming around HPV particles, suggesting that HPV transiently modulates PML NB composition. In contrast to large DNA viruses, which encode factors capable of reorganizing PML NBs, HPV seems to take advantage of the disassembly occurring at the onset of mitosis. As such, it utilizes well-established cellular pathways to orchestrate the regulation of viral transcription during the immediate early events of the viral life cycle.

## Introduction

Promyelocytic leukemia (PML) nuclear bodies (NBs) are highly dynamic nuclear structures that have been associated with numerous cellular processes, including apoptosis, transcriptional regulation, and innate and intrinsic immune responses [1]. While their size and number of residing proteins vary according to the cell condition, the main component of PML NBs is PML protein [2]. It is present in seven isoforms that constitute the main scaffold of PML NBs, with the exception of PML VII that lacks a nuclear localization signal and remains in the cytosol [3]. PML protein is required for the formation and stability of PML NBs, as cells knocked down for PML protein fail to form these structures [4]. SUMOylation of PML protein with SUMO-1 and SUMO-2 is necessary for this process and SUMOylated PML proteins then recruit other PML NB-residing proteins that are either SUMOylated themselves or contain SUMO interacting motifs [2,4,5,6,7]. Transcriptional repressors Sp100 and Daxx are two additional proteins that permanently reside in PML NBs [1]. PML NBs are modified during cell cycle progression [1,8,9]. They disassemble upon the onset of mitosis and PML protein forms large aggregates in the cytosol, also referred to as mitotic accumulations of PML proteins (MAPPs). MAPPs do not contain Sp100 or Daxx but only de-SUMOylated PML protein and are recycled after completion of mitosis. Following nuclear envelope reformation, released PML protein molecules are translocated back into the nucleus to form new PML NBs in the daughter cell nuclei and recruit other proteins.

Despite extensive research on the role of PML NBs, their specific function in the cell remains unclear. However, they have been shown to be involved in innate and intrinsic immunity as both repressors of viral gene expression and coregulators of the type I interferon pathway [10]. Consequently, many DNA viruses, such as herpes simplex 1 (HSV-1), human cytomegalovirus (HCMV), simian virus 40 (SV40) and adenovirus 5 (ADV5), target PML NBs during primary infection and induce the reorganization and degradation of the residing proteins, including PML protein and Sp100 [11–15]. Specifically, HSV-1 and ADV5 encode early immediate proteins, ICP0 and E1A-13S, respectively, which target PML protein isoforms for degradation and thus enhances viral gene expression [15,16]. Similarly, HCMV targets Sp100 for degradation through its immediate early protein IE1 to prevent their transcriptional repression activity and enhance the early stages of infection [14]. It is thought that PML NBs are sensors of DNA/protein complexes and are thus recruited to virally-induced foci [17,18]. In the case of HSV-1 infection, PML NBs have recently been shown to be recruited to incoming HSV-1 genomes following nuclear delivery [18,19]. Furthermore, high-resolution microscopy showed that PML NB-residing proteins engulfed viral genomes shortly after nuclear entry. As SUMOylation and SUMO interaction are critical for the formation and dynamics of PML NBs, HSV-1 ICP0 is thought to target PML protein through SUMO interaction or recognition of their SUMO-1 conjugation motifs [16,20].

Similar to most other DNA viruses, papillomaviruses (PVs) associate with PML NBs at several stages of their life cycle. PV genomes along with minor capsid protein L2 have been observed to associate with PML NBs after infectious delivery into the nucleus of target cells. L2 protein also localizes to PML NBs in natural productive lesions, although transiently, and when over-expressed in cell culture [21–24]. However, while PML NBs restrict gene expression of most viruses, PVs, such as bovine papillomavirus 1 (BPV1) and human papillomavirus (HPV) types 16 and 18, have been shown to require PML protein for efficient transcription [25–27]. Transcription driven by both PV and heterologous promoters delivered by PV particles was repressed in the absence of PML protein, suggesting that PML protein does not function in a promoter-specific manner [25]. In addition, Sp100 was shown to restrict HPV18 transcription and replication [26,28]. These findings suggest that PML NBs may play an important role in the regulation of the PV life cycle.

Following infectious entry, the HPV capsid uncoats within acidified endocytic vesicles. This is facilitated by host cell cyclophilin which allows for the partial dissociation of the major capsid protein L1 from the minor capsid protein L2, which remains in complex with the viral genome [29–31]. While most of the L1 protein appears to be degraded in the late endosome, a subset of L1 protein, likely arranged as capsomeres, remains associated with the viral genome [30–32]. L2 protein assumes a transmembranous configuration, which is promoted by a newly described chaperone function of γ-secretase [33]. A putative transmembrane region spanning from residues 45 to 65 separates a small luminal domain from the large carboxy-terminal region that can interact with cytosolic factors, including the machinery mediating retrograde transport along microtubules (MTs) towards the *trans*-Golgi network (TGN) [34,35]. Prior to associating with PML NBs, the HPV genome needs to be delivered to the nucleus. Rather than utilizing nuclear pores and the nuclear import machinery, HPV takes advantage of nuclear envelope breakdown during mitosis to gain access to the nucleus [36,37]. HPV-harboring vesicles likely rely on L2 protein, which retains its transmembranous configuration during vesicular mitotic transport, to interact with motor proteins, such as dynein and kinesins, for transport along astral and spindle MTs, respectively [32,38–40]. Surprisingly, the viral genome resides within the transport vesicle in the nucleus for several hours after completion of mitosis and nuclear envelope reformation, resulting in delayed transcription when compared to delivery of naked DNA [25,30].

More recently, we reported that viral pseudogenomes delivered by HPV16 particles were lost after successful nuclear delivery in the absence of PML protein in the spontaneously immortalized HaCaT keratinocytes but not in HPV18 transformed HeLa cells [27]. Viral genome loss in HaCaT cells was prevented by inhibitors of the Jak/Stat signaling axis, although transcription was not restored. These findings pointed towards a protective role of PML protein in the immediate early stages of the HPV life cycle. Thus, we were prompted to pose the following questions: 1) when does the association of viral genome with PML protein and other PML NB-residing proteins occur; 2) which viral factors may play a role mediating this interaction during infection; and 3) whether viral genomes target preformed PML NBs or rather PML NB-residing proteins are recruited to incoming HPV genomes. Given that DNA successfully delivered to the nucleus by HPV particles is lost in the absence of PML protein, we hypothesized that PML protein is recruited to HPV-harboring transport vesicles prior to release from this membrane-bound environment and that likely L2 protein is mediating this association. L2 protein harbors a SUMO conjugation domain on its N-terminus, as well as one highly conserved SUMO interactive motif (SIM) and two additional putative SIMs on its C-terminus, which we hypothesized might be involved in recruitment of PML protein [24,41]. We also hypothesized that the recruitment of Sp100 is delayed and occurs after release of the viral genome from the transport vesicle. Herein, we utilized differential staining of viral pseudogenomes in combination with high-resolution immunofluorescence to determine the spatio-temporal recruitment of PML protein, Sp100, and SUMO-1 to incoming viral genomes during infectious entry and establish an order of events following nuclear delivery of HPV genomes [30,32].

## Results

### Estimating age of interphase cells in asynchronous cells

**Fig 1:**
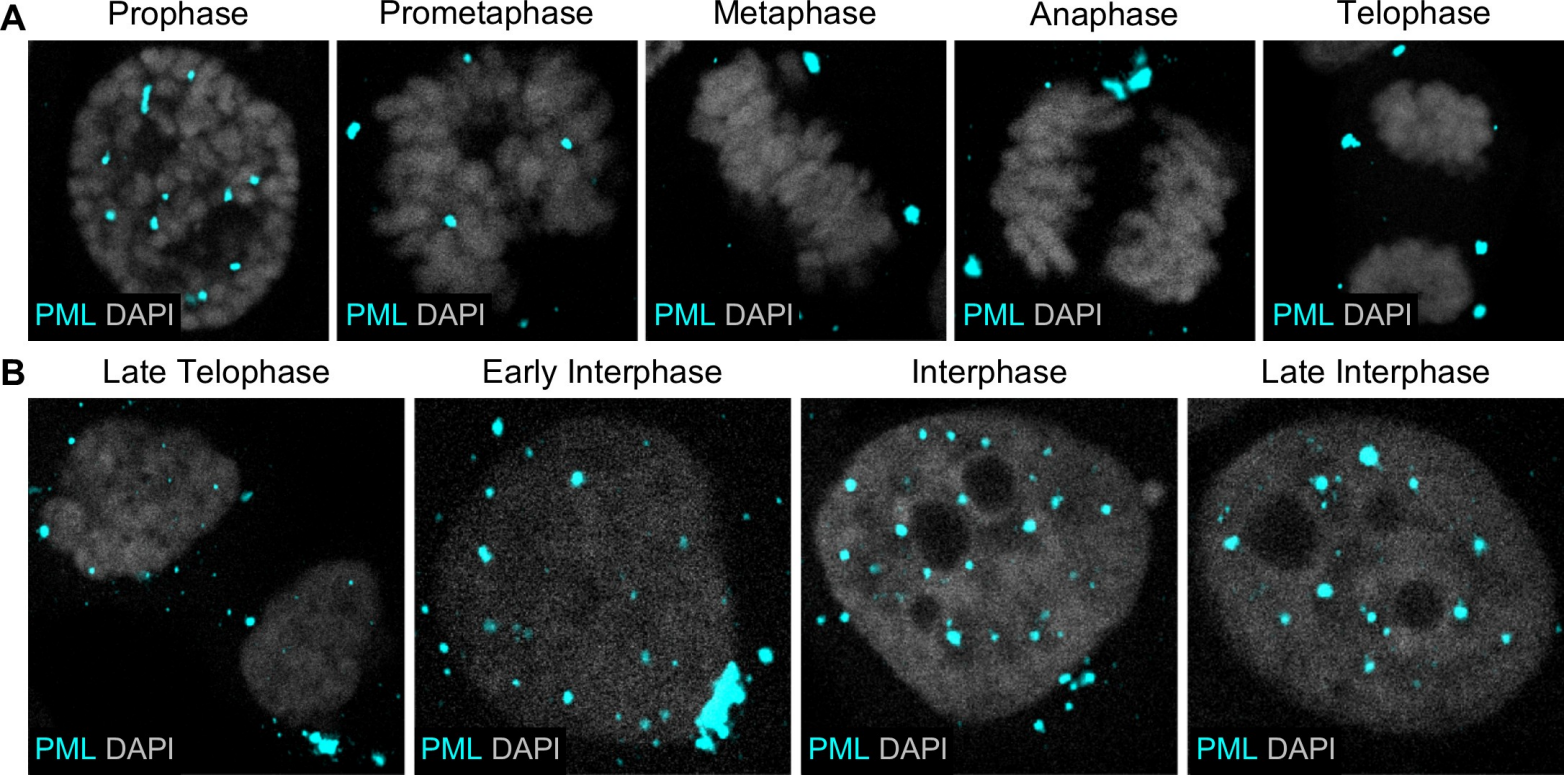
PML protein dynamics is used to approximate time during and after mitosis. HaCaT cells were grown on glass coverslips, then fixed, permeabilized, incubated with rabbit anti-PML protein antibody (cyan), and mounted with DAPI (white). Confocal images show cells in all stages of mitosis (A) and end of mitosis and stages of interphase (B).

To investigate the order of events following nuclear delivery of viral genomes, we needed to estimate the amount of time that has passed throughout mitosis and interphase. To achieve this, we observed the morphology of the nucleus and the localization of PML protein by immunofluorescent microscopy (Fig 1). During mitosis, PML protein forms MAPPs that are observed around mitotic chromosomes at all stages of mitosis (Fig 1A) [8,9,42]. Using DAPI staining, we define late telophase as cells exhibiting decondensing DNA and reforming nucleus, as well as the presence of PML protein aggregates (Fig 1B). As MAPPs translocate back into the nucleus after nuclear envelope reformation, early interphase cells harbor large cytosolic aggregates of PML protein and a few, typically small, PML protein foci in the newly formed nucleus. The number and size of MAPPs decrease, while the number and size of PML protein foci inside the nucleus increase, throughout interphase. To estimate how much time has elapsed after the completion of mitosis, we counted the number of nucleoli, as it is inversely correlated with time, a method we have used in our previously published work [30,43]. Therefore, we estimated that 7+ nucleoli are present in the nucleus of early interphase cells, while late interphase cells are characterized by 1–6 nucleoli.

### PML protein is recruited to incoming viral genomes

**Fig 2:**
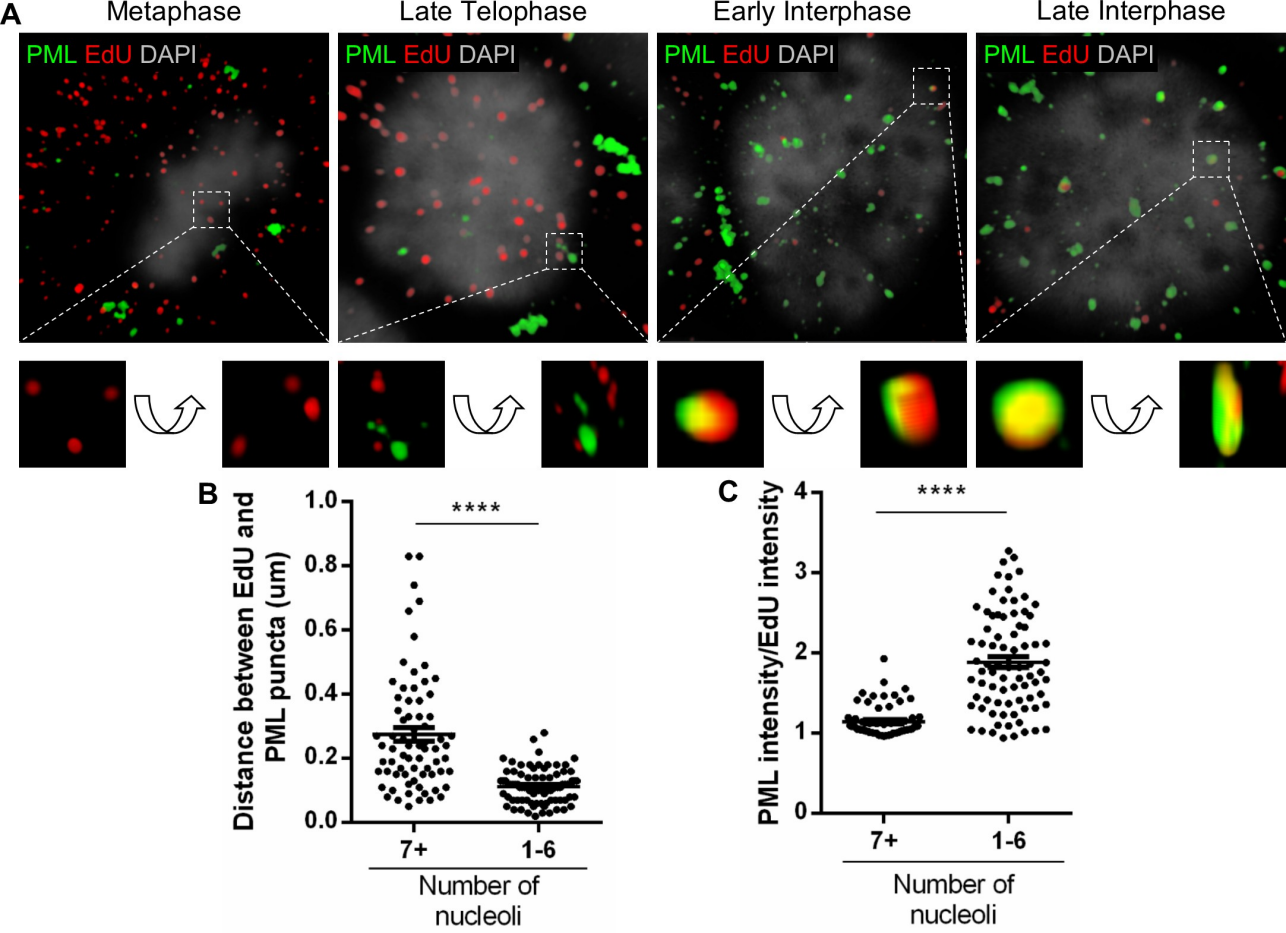
PML protein encompasses incoming viral genomes only after nuclear delivery. HaCaT cells were infected with EdU-labeled PsVs for 24 h, fixed, permeabilized, and treated with Click-iT reaction buffer with AF555 dye to stain EdU-labeled pseudogenomes (red). Next, cells were incubated with mouse anti-PML protein antibody (green) and mounted in DAPI (white). (A) Images show 3D reconstruction of high resolution z-stacks and close-up images were rotated at a 45° angle on the x, y, z axes in the 3D-rendered images. (B) Distance (μm) between center of EdU and center of PML protein puncta was measured in NIS Elements. (C) PML and EdU mean intensity were measured in NIS Elements, normalized to ROI area, and the ratio was calculated. (B and C) Nucleoli were also counted: 7+ nucleoli corresponds to early interphase, 1–6 to late interphase. Results are shown as average of 2 independent experiments and SEM, with 30–50 cells of each condition (early and late interphase cells) collected in z-stacks spanning the whole nucleus. (B) 7+ nucleoli: 0.275 μm ± 0.021 μm; 1–6 nucleoli: 0.113 μm ± 0.006 μm. (C) 7+ nucleoli: 1.146 ± 0.025; 1–6 nucleoli: 1.888 ± 0.069. *p* value was determined using Student’s *t*-test comparing Early to Late Interphase. ****: *p* < 0.0001.

**Fig 3:**
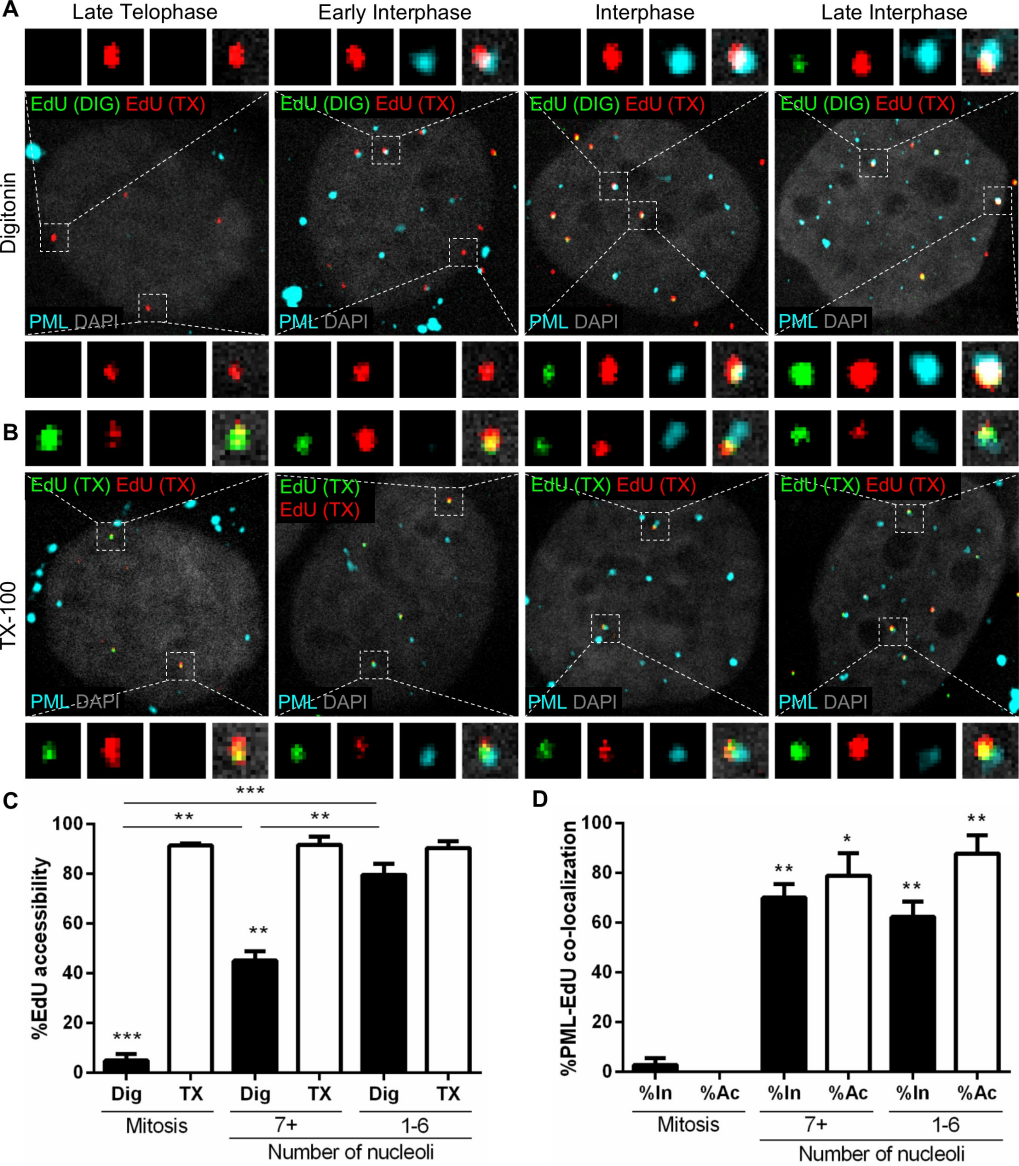
Viral genome associates with PML protein while still inaccessible to small molecular dyes. (A and B) HaCaT cells were infected with EdU-labeled PsVs for 24 h, fixed, permeabilized with 0.625 μg/mL digitonin (A) or 0.5% TX-100 (B), and treated with AF555 (green) in Click-iT reaction buffer. Next, the cells were permeabilized again with 0.5% TX-100 and treated with AF647 (red) in Click-iT reaction buffer. Lastly, cells were incubated with rabbit anti-PML protein antibody (cyan) and mounted with DAPI (white). (C and D) Percent accessibility of viral genome (C) was determined by manually counting the number of red only (inaccessible [In]) or red/green (accessible [Ac]) EdU puncta over the total number of EdU puncta associated with condensed chromosomes in mitotic cells or nuclear-localized in interphase cells in digitonin- or TX-100-permeabilized cells. Nucleoli were also counted: 7+ nucleoli corresponds to early interphase, 1–6 to late interphase. Co-localization of PML protein and EdU (D) was quantified by manually counting the number of EdU puncta that co-localized with PML protein signal over the total number of accessible or inaccessible EdU puncta associated with condensed chromosomes in mitotic cells or nuclear-localized in interphase in digitonin-permeabilized cells. Results are shown as average of 3 independent experiments and SEM, with 30–50 cells of each condition (mitotic, early and late interphase cells) collected in z-stacks spanning the whole nucleus. (C) Mitosis: Dig: %Ac = 4.90% ± 2.55%; TX: %Ac = 91.37% ± 0.77%. 7+ nucleoli: Dig: %Ac = 45.05% ± 3.80%; TX: %Ac = 91.62% ± 3.38%. 1–6 nucleoli: Dig: %Ac = 79.55% ± 4.38%; TX: %Ac = 90.35% ± 2.63%. (D) Mitosis: %In at PML = 2.73% ± 2.73%; %Ac at PML = 0% ± 0%. 7+ nucleoli: %In at PML = 70.09% ± 5.43%; %Ac at PML = 78.85% ± 9.05%. 1–6 nucleoli: %In at PML = 62.22% ± 6.19%; %Ac at PML = 76.43% ± 7.57%. *p* value was determined using Student’s *t*-test comparing Dig to TX and Dig to Dig (C) or Interphase to Mitosis (D). ***: *p* < 0.001. **: *p* < 0.01. *: *p* < 0.05. ns: *p* > 0.05.

Although the association of HPV genomes with PML protein has been known for decades, how it occurs is still unclear [22]. It has been assumed that incoming viral genomes are targeted to preformed PML NBs rapidly after nuclear delivery [22,25], despite the now known dynamics of PML protein [8,9]. However, in HSV-1 infection, PML protein was shown to be recruited to and engulf incoming viral genomes following nuclear entry by high resolution microscopy [19]. Furthermore, our previously published findings suggest that PML protein provides a protective environment for the viral genome [27]. Therefore, we wanted to determine whether PML protein is recruited towards viral genomes or vice versa and to visualize the architecture of this association. We acquired high resolution images of HaCaT cells infected with HPV16 pseudovirions (PsVs) harboring an EdU-labeled pseudogenome after immunofluorescent staining. Images are z-stacks combined with 3D reconstruction for PML protein (green) and EdU-labeled viral pseudogenomes (red) (Fig 2A). While previous confocal microscopy could only show EdU puncta adjacent to PML protein [25,27], high resolution microscopy allows us to observe the structure of PML protein in association with incoming viral genomes in the nucleus of infected cells. As expected, PML protein aggregates in cytosolic MAPPs in mitotic cells, whereas the EdU-labeled pseudogenomes are present throughout the cell with some in the vicinity of mitotic chromosomes as previously reported [30,36]. Majority of EdU puncta did not co-localize with PML protein aggregates during mitosis (for quantification, see Fig 3D), such as metaphase or late telophase, unlike previously reported [44]. However, we observed EdU puncta co-localizing with PML protein foci of different sizes in early interphase cells, which is then engulfed in the later interphase cells. In order to quantify these observations, we measured the distance between the center of EdU puncta and the center of PML protein puncta in early (7+ nucleoli) or late (1–6 nucleoli) interphase and found that PML protein is very closely co-localizing with EdU in late interphase cells, while the distance is very variable and overall greater in early interphase cells (Fig 2B). In addition, as a measurement of engulfment, we calculated the ratio of PML intensity over EdU intensity, each normalized to the area of co-localizing foci (Fig 2C). While the intensity of EdU puncta remained similar throughout, the intensity of PML puncta significantly increased in late interphase as we observe entrapment of EdU signal. Taken together, these data indicate that PML protein targets incoming viral genomes and forms around them, rather than viral genomes being recruited to preformed PML protein structures.

### PML protein recruitment occurs prior to the viral genome becoming accessible within the nucleus

We previously described that incoming viral genomes are lost in cells depleted for PML protein, implying that PML protein provides a protective environment for the viral genomes. If this is the case, we would predict that PML protein accumulates around incoming pseudogenomes before the release from the transport vesicles. To test this assumption, we employed a differential staining technique that has been previously described by our lab [30,32]. HaCaT cells were infected with EdU-labeled PsVs for 24 h. Following fixation, cells were permeabilized with a low concentration of digitonin, which only permeabilizes cholesterol-rich membranes, such as the plasma membrane and endocytic vesicles directly derived from the plasma membrane. Next, the cells were subjected to the Click-iT reaction using AlexaFluor (AF) 555 as reactive dye to stain the viral genome (green). Subsequently, cells were completely permeabilized with Triton X-100 (TX-100) and subjected to another round of Click-iT reaction, this time using AF647 dye to stain the viral genome (red). Only EdU-labeled genomes either present on the cell surface, in early endocytic vesicles, or after egress from the endocytic compartment will be stained with AF555, whereas all genomes will be stained with AF647 after TX-100 permeabilization. As a positive control, we treated cells with TX-100 instead of digitonin prior to the first staining for total permeabilization (S1 Fig, Fig 3B). To control for intracellular membrane integrity, we tested the reactivity of an antibody recognizing a luminal epitope of TGN46 (cyan) in digitonin- and TX-100-treated cells (S1 Fig). We observed that the luminal epitope of TGN46 was recognized in TX-100-treated cells, but not in digitonin-treated cells, suggesting that the plasma membrane but not internal membranes were permeabilized with the low concentration of digitonin. Representative images of infected HaCaT cells in various stages of mitosis demonstrate the inaccessibility of pseudogenomes to AF555 after digitonin but not TX-100 permeabilization (S1 Fig). We combined differential staining of EdU-labeled pseudogenomes with immunofluorescent staining for PML protein (cyan) and quantified the presence of PML protein as a function of genome accessibility in different phases of the cell cycle (Fig 3). Once again, we observed essentially no co-localization of PML protein with EdU in late telophase cells and it was not until after mitosis was completed that viral genomes were shown co-localizing with PML protein (Fig 3A and 3B). In early interphase cells, PML protein co-localized with nuclear-localized EdU puncta that were inaccessible to AF555 after digitonin permeabilization, whereas EdU puncta were accessible to both dyes and co-localized with PML protein in late interphase cells. We quantified the number of single red (inaccessible) or dual green/red (accessible) EdU puncta in mitosis and early (7+ nucleoli) and late (1–6 nucleoli) interphase (Fig 3C). Chromosome-localized EdU puncta were 95% inaccessible in mitosis (5% accessible), and become more accessible over time after completion of mitosis in digitonin-treated cells (45% and 80% accessible EdU in early and late interphase, respectively). In the TX-100-treated control cells, EdU was consistently 95% accessible throughout the cell cycle (Fig 3C). These results are consistent with our published findings suggesting that accessibility of the viral genome is delayed after completion of mitosis [30]. Next, we quantified the number of inaccessible (In) or accessible (Ac) EdU puncta that co-localized with PML protein in mitosis, early interphase (7+ nucleoli), and late interphase (1–6 nucleoli) in digitonin-treated cells (Fig 3D). During mitosis, EdU puncta did not co-localize with PML protein, which was visible as large cytosolic aggregates. In interphase cells, accessible EdU puncta largely co-localized with PML protein (79% and 76% in early and late interphase, respectively). Interestingly, we observed 70% of inaccessible EdU puncta co-localized with PML protein in early interphase. This implies that EdU puncta co-localize with PML protein while the viral genomes are still inaccessible immediately after completion of mitosis and remains associated to a comparable level in late interphase (62%). The differences in PML protein co-localization with inaccessible and accessible EdU puncta in early and late interphase cells were not determined to be statistically significant, thereby suggesting that EdU and PML protein associate in early interphase and remain associated throughout interphase. Taken together, these data suggest that, as PML protein translocates back into the nucleus following completion of mitosis, PML protein is subsequently recruited to incoming viral genomes when still inaccessible and they remain associated as the cell progresses through interphase and viral genomes become accessible.

### Timing of SUMO-1 recruitment to viral genomes is similar to PML protein

**Fig 4:**
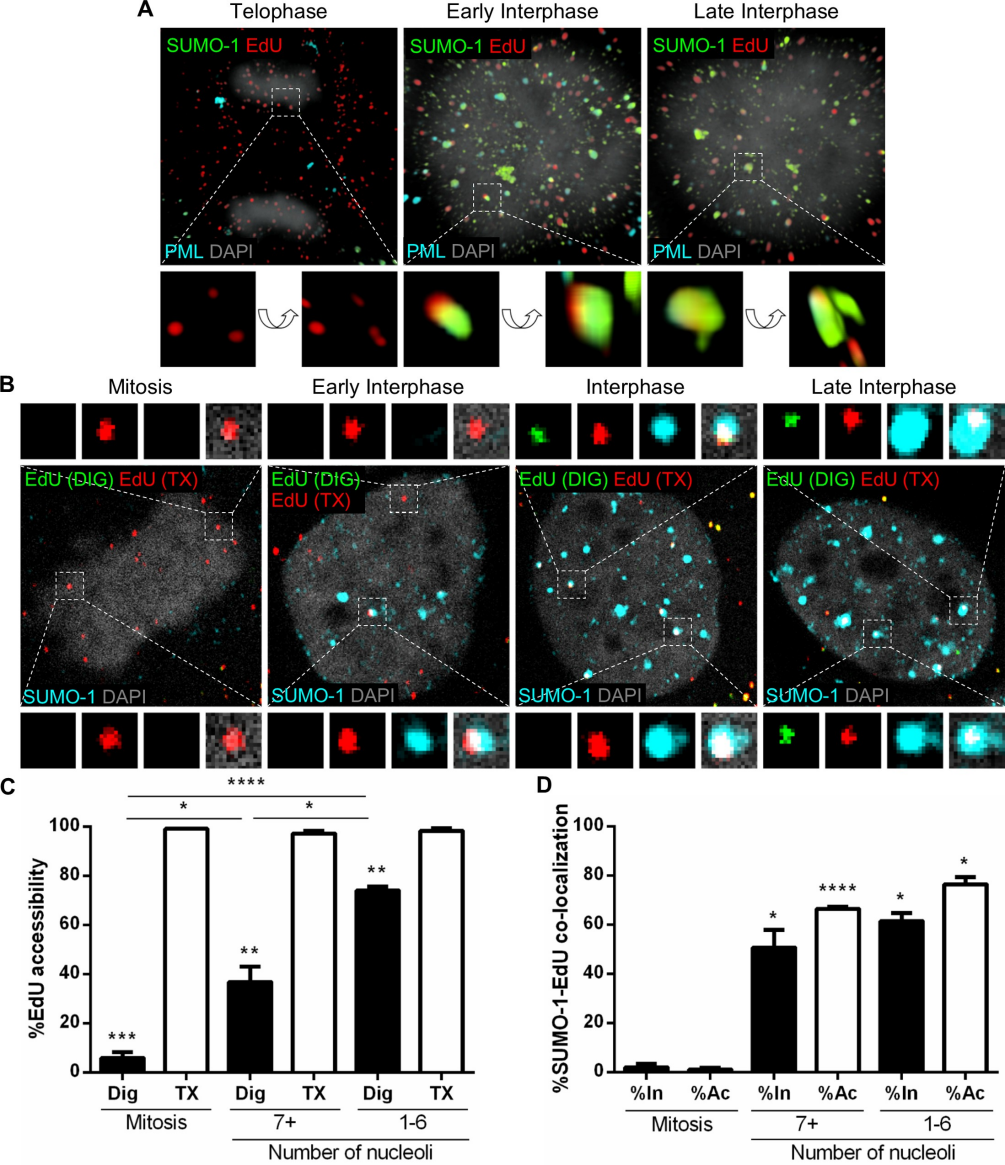
Viral genome associates with SUMO-1 while still inaccessible to small molecular dyes. (A) HaCaT cells were infected with EdU-labeled PsVs for 24 h, fixed, permeabilized, and treated with AF555 (red) in Click-iT reaction buffer. Next, cells were incubated with rabbit anti-SUMO-1 antibody (green) and mouse anti-PML protein antibody (cyan) and mounted in DAPI (white). Images show 3D reconstruction of high resolution z-stacks. Close-up images were rotated at a 45° angle on the x, y, z axes in the 3D-rendered images. (B) HaCaT cells were infected with EdU-labeled PsVs for 24 h, fixed, permeabilized with 0.625 μg/mL digitonin and treated with AF555 (green) in Click-iT reaction buffer. Next, the cells were permeabilized again with 0.5% TX-100 and treated with AF647 (red) in Click-iT reaction buffer. Lastly, cells were incubated with rabbit anti-SUMO-1 antibody (cyan) and mounted with DAPI (white). (C and D) Percent accessibility of viral genome (C) was determined by manually counting the number of red only (inaccessible [In]) or red/green (accessible [Ac]) EdU puncta over the total number of EdU puncta associated with condensed chromosomes in mitotic cells or nuclear-localized in interphase cells in digitonin- or TX-100-permeabilized cells. Nucleoli were also counted: 7+ nucleoli corresponds to early interphase, 1–6 to late interphase. Co-localization of SUMO-1 and EdU (D) was quantified by manually counting the number of EdU puncta that co-localized with SUMO-1 signal over the total number of accessible or inaccessible EdU puncta associated with condensed chromosomes in mitotic cells or nuclear-localized in interphase in digitonin-permeabilized cells. Results are shown as average of 3 independent experiments and SEM, with 30–50 cells of each condition (mitotic, early and late interphase cells) collected in z-stacks spanning the whole nucleus. (C) Mitosis: Dig: %Ac = 5.85% ± 2.41%; TX: %Ac = 99.09% ± 0.12%. 7+ nucleoli: Dig: %Ac = 36.78% ± 6.21%; TX: %Ac = 97.22% ± 1.10%. 1–6 nucleoli: Dig: %Ac = 74.00% ± 1.73%; TX: %Ac = 98.30% ± 1.18%. (D) Mitosis: %In at SUMO-1 = 1.99% ± 1.48%; %Ac at SUMO-1 = 1.09% ± 0.63%. 7+ nucleoli: %In at SUMO-1 = 50.73% ± 7.14%; %Ac at SUMO-1 = 66.48% ± 0.77%. 1–6 nucleoli: %In at SUMO-1 = 61.45% ± 3.27%; %Ac at SUMO-1 = 76.47% ± 2.79%. *p* value was determined using Student’s *t*-test comparing Interphase to Mitosis. ****: *p* < 0.0001. ***: *p* < 0.001. **: *p* < 0.01. *: *p* < 0.05. ns: *p* > 0.05.

PML protein is SUMOylated and interacts with and recruits other proteins by non-covalent SUMO interactions, mainly through SUMO-1, which is essential for PML NB formation, stability, and localization [2,4]. Therefore, we sought to examine the recruitment of SUMO-1 to incoming viral genomes. To address this, we performed immunofluorescent staining on EdU-labeled PsV-infected HaCaT cells to detect EdU-labeled viral pseudogenomes (red), PML protein (cyan), and SUMO-1 (green) and acquired high resolution images of z-stacks combined by 3D reconstruction (Fig 4A). We observed very limited detection of SUMO-1 aggregates in cells undergoing mitosis, with little to no co-localization with PML protein; whereas, following the completion of mitosis, SUMO-1 was detected co-localizing with PML protein in the nucleus of interphase cells. In addition, EdU puncta co-localized with these SUMO-1/PML protein foci in the nucleus of interphase cells. SUMO-1 was also observed encompassing the EdU signal in a similar manner as PML protein alone previously was (Fig 2). Next, we investigated SUMO-1 co-localization as a function of viral genome accessibility. We combined immunofluorescent staining of SUMO-1 with the same differential staining technique described in S1 Fig and Fig 3. HaCaT cells were infected with EdU-labeled PsVs and differentially stained to detect inaccessible (red) and accessible (green/red) EdU-labeled pseudogenomes along with SUMO-1 (cyan) in mitotic and interphase cells (Fig 4B). EdU accessibility was quantified in mitosis, early interphase (7+ nucleoli), and late interphase (1–6 nucleoli) in digitonin- and TX-100-treated cells. Just like we observed in Fig 3, we reproduced the same pattern of EdU genome accessibility throughout the cell cycle (Fig 4C). Next, we quantified the number of inaccessible (In) or accessible (Ac) EdU puncta that co-localized with SUMO-1 in mitosis, early interphase (7+ nucleoli), and late interphase (1–6 nucleoli) in digitonin-treated cells (Fig 4D). Not surprisingly, SUMO-1 co-localization with EdU puncta was very similar to PML protein in Fig 3D. EdU puncta did not co-localize with SUMO-1 during mitosis (2% and 1% of inaccessible and accessible EdU, respectively). However, 51% of inaccessible EdU puncta co-localize with SUMO-1 in early interphase and 61% in late interphase. Accessible EdU puncta also largely co-localize with SUMO-1 in early and late interphase (66% and 76%, respectively). Taken together, these data suggest that SUMO-1 is recruited to incoming viral genomes prior to becoming accessible, likely along with PML protein.

### L1 protein co-localization with PML protein decreases with interphase progression while L2 protein remains at PML protein with the viral genome

**Fig 5:**
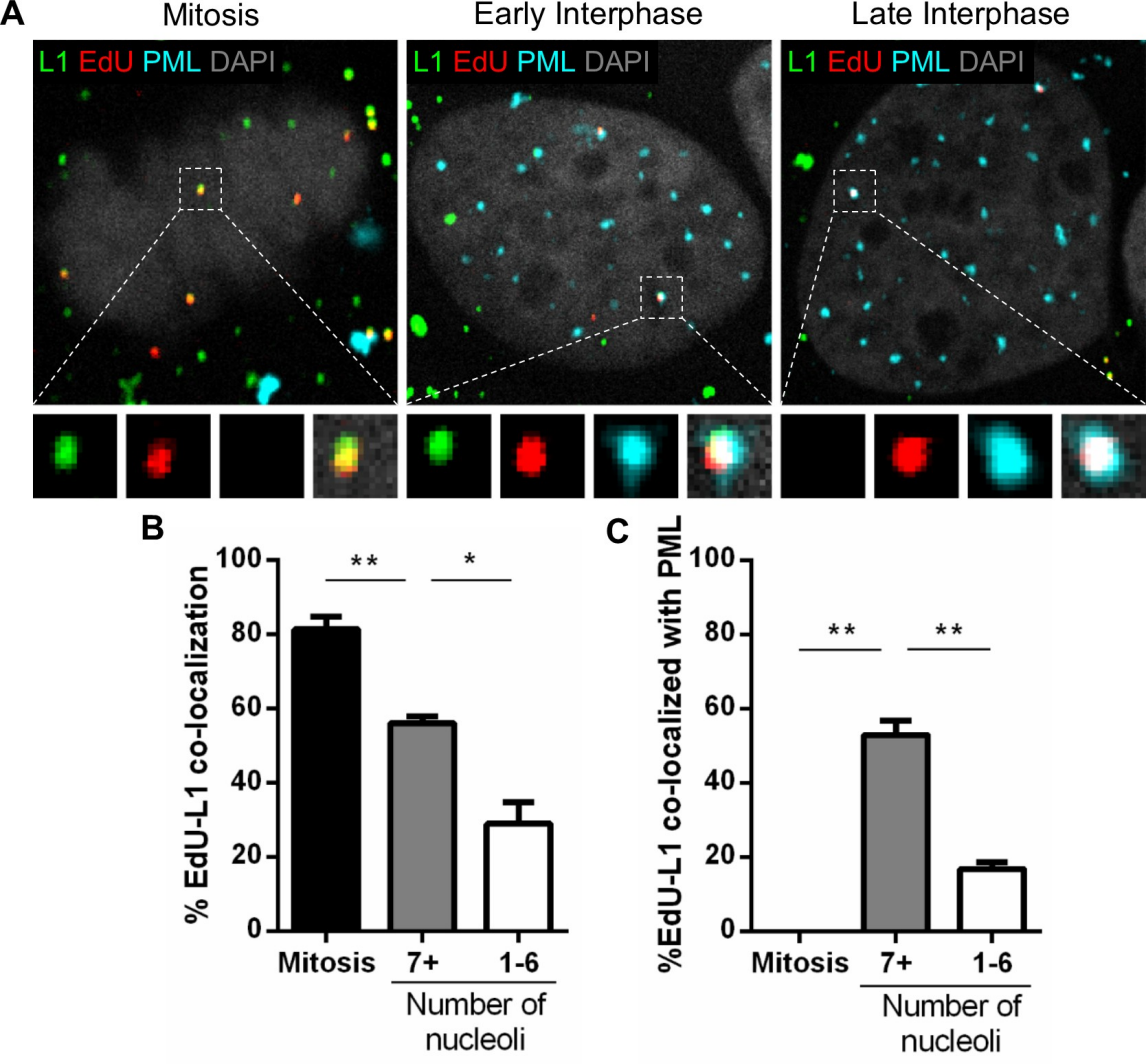
PML protein is recruited to the viral genome prior to the loss of L1 protein in late interphase. (A) HaCaT cells were infected with EdU-labeled PsVs for 24 h, fixed, permeabilized, and treated with AF555 (red) in Click-iT reaction buffer. Next, the cells were incubated with rabbit anti-PML protein antibody (cyan) and mouse 33L1-7 antibody (green) for specific detection of L1 protein and mounted with DAPI (white). (B) Percent co-localization of EdU and L1 was determined by manually counting the number of EdU puncta and L1 puncta (red/green) over the total number of EdU puncta (red only) associated with condensed chromosomes in mitotic cells or nuclear-localized in interphase cells. Nucleoli were also counted: 7+ nucleoli corresponds to early interphase, 1–6 to late interphase. (C) Percent co-localization of EdU with PML protein was determined by manually counting the number of EdU-L1 puncta (red/green) co-localizing with PML protein signal over the total number of EdU puncta (red only) co-localizing with PML protein associated with condensed chromosomes in mitotic cells or nuclear-localized in interphase cells. Results are shown as average of 3 independent experiments and SEM, with 30–50 cells of each condition (mitotic, early and late interphase cells) collected in z-stacks spanning the whole nucleus. (B) Mitosis: %EdU-L1 = 81.48% ± 3.41%. 7+ nucleoli: %EdU-L1 = 56.19% ± 1.72%. 1–6 nucleoli: %EdU-L1 = 29.01% ± 5.87%. (C) Mitosis: %EdU-L1 at PML = 0% ± 0%. 7+ nucleoli: %EdU-L1 at PML = 52.94% ± 3.86%. 1–6 nucleoli: %EdU-L1 at PML = 16.79% ± 1.74%. *p* value was determined using Student’s *t*-test comparing Interphase to Mitosis. ****: *p* < 0.0001. ns: *p* > 0.05.

Our recently published work suggested that a subset of the L1 protein traffics and is delivered into the nucleus along with the L2/viral genome complex [31]. Our data also indicated that L1 protein resides within the transport vesicle during trafficking and is lost when viral genomes become accessible in the nucleus. Therefore, we hypothesized that PML protein would associate with L1 together with viral genomes in early interphase. To test this, we performed immunofluorescent staining on HaCaT cells infected with EdU-labeled PsVs (Fig 5). Representative images of infected HaCaT cells in mitosis, early, and late interphase stained for PML protein (cyan), EdU-labeled pseudogenomes (red), and L1 protein (green) are displayed (Fig 5A). We quantified the total number of chromosome-associated or nuclear EdU puncta co-localizing with L1 protein in following phases of the cell cycle: mitosis, early interphase (7+ nucleoli), and late interphase (1–6 nucleoli) (Fig 5B). During mitosis, the majority of EdU puncta co-localized with L1 (81%). The L1 signal is still present with EdU in early interphase (56%) but is dramatically reduced in late interphase (30%), which is consistent with our published results [31]. Next, we quantified the number of EdU-L1 puncta co-localizing with PML protein during mitosis, early (7+ nucleoli) and late (1–6 nucleoli) interphase (Fig 5C). Once again, PML protein did not co-localize with EdU puncta during mitosis (0%). However, EdU/L1 signal did co-localize with PML protein in the nucleus of early interphase cells (53%). In late interphase, while EdU puncta remained co-localized with PML protein, L1 signal is lost (17%). Taken together, these data suggest that L1 remains associated with the viral genome as PML protein is recruited in early interphase but is lost in later stages of interphase, which corresponds to when viral genomes become accessible.

**Fig 6:**
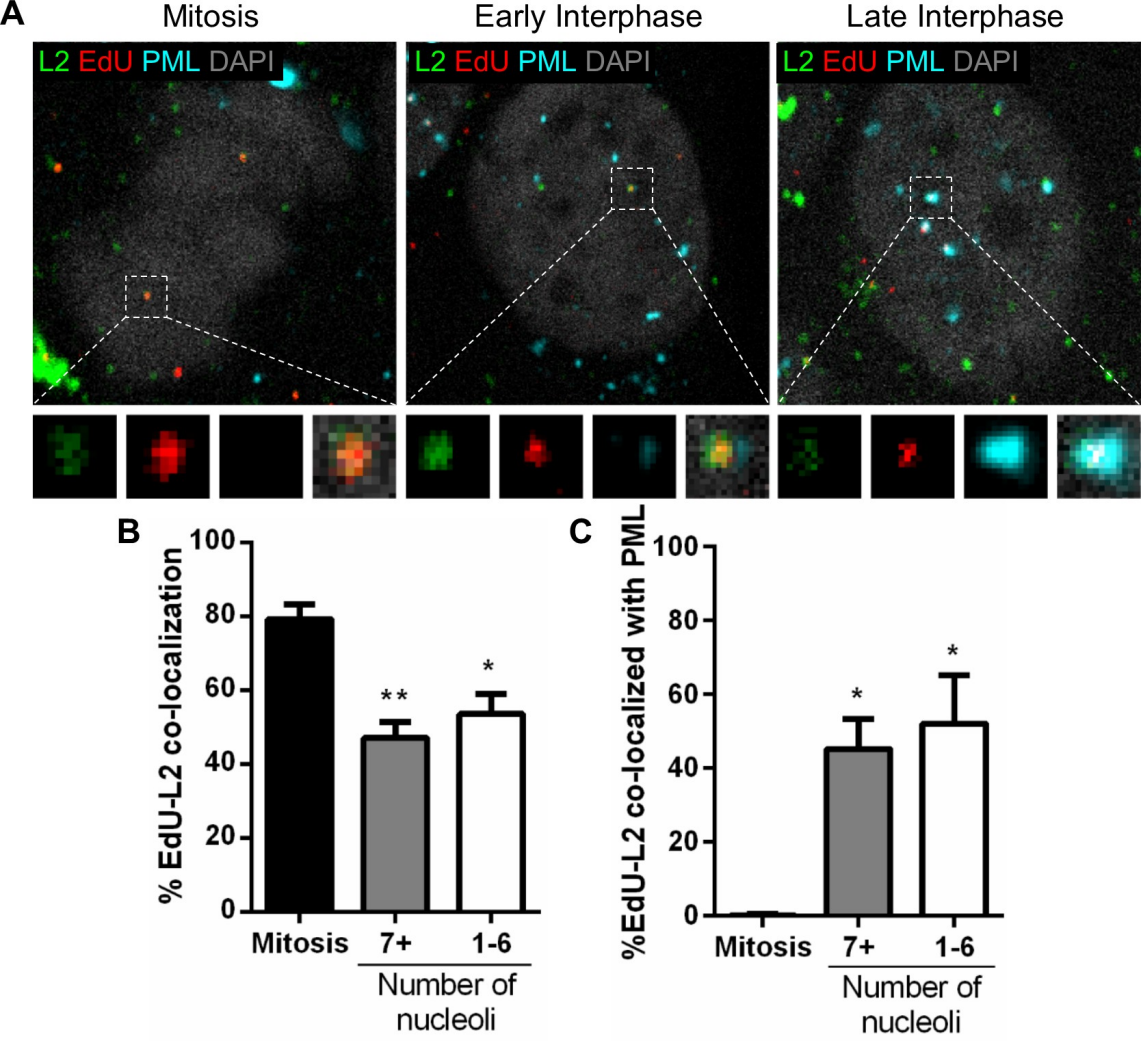
L2 protein remains associated with the viral genome in late interphase. (A) HaCaT cells were infected with EdU-labeled PsVs for 24 h, fixed, permeabilized, and treated with AF555 (red) in Click-iT reaction buffer. Next, the cells were incubated with rabbit anti-PML protein antibody (cyan) and mouse 33L2-1 antibody (green) for specific detection of L2 protein and mounted with DAPI (white). (B) Percent co-localization of EdU and L2 was determined by manually counting the number of EdU puncta and L2 puncta (red/green) over the total number of EdU puncta (red only) associated with condensed chromosomes in mitotic cells or nuclear-localized in interphase cells. Nucleoli were also counted: 7+ nucleoli corresponds to early interphase, 1–6 to late interphase. (C) Percent co-localization of EdU with PML protein was determined by manually counting the number of EdU-L2 puncta (red/green) co-localizing with PML protein signal over the total number of EdU puncta (red only) co-localizing with PML protein associated with condensed chromosomes in mitotic cells or nuclear-localized in interphase cells. Results are shown as average of 3 independent experiments and SEM, with 30–50 cells of each condition (mitotic, early and late interphase cells) collected in z-stacks spanning the whole nucleus. (B) Mitosis: %EdU-L2 = 79.22% ± 4.06%. 7+ nucleoli: %EdU-L2 = 47.11% ± 4.29%. 1–6 nucleoli: %EdU-L2 = 53.59% ± 5.42%. (C) Mitosis: %EdU-L2 at PML = 0.22% ± 0.22%. 7+ nucleoli: %EdU-L2 at PML = 45.20% ± 4.69%. 1–6 nucleoli: %EdU-L2 at PML = 52.11% ± 7.60%. *p* value was determined using Student’s *t*-test comparing Interphase to Mitosis. **: *p* < 0.01. *: *p* < 0.05.

In contrast to L1, we had showed that L2 proteins remained with the viral genome for a longer period of time, even after viral genomes become accessible [31]. Logically, we sought to investigate whether L2 remained at PML NBs along with the viral genomes as interphase progresses and performed the same analysis as for L1 protein (Fig 6). Representative images of infected HaCaT cells in mitosis, early, and late interphase stained for PML protein (cyan), EdU-labeled pseudogenomes (red), and L2 protein (green) are displayed (Fig 6A). We quantified the total number of chromosome-associated or nuclear EdU puncta co-localizing with L2 protein in the different phases of the cell cycle (Fig 6B). Although there was a decrease in the amount of L2 co-localized with EdU between mitosis and early interphase, similarly to what was observed with L1, the L2 signal remained constant with about 50% L2 viral genomes after completion of mitosis and was still present in late interphase. Then, we quantified the number of EdU-L2 co-localizing with PML protein in the same conditions as previously described (Fig 6C). As expected, L2 also remained with EdU co-localized with PML protein even in late interphase cells. Taken together, these data suggest that, while L1 is lost when viral genomes become accessible in later stages of interphase, L2 remains associated with the now accessible viral genomes.

### Recruitment of Sp100 is delayed compared to PML protein

**Fig 7:**
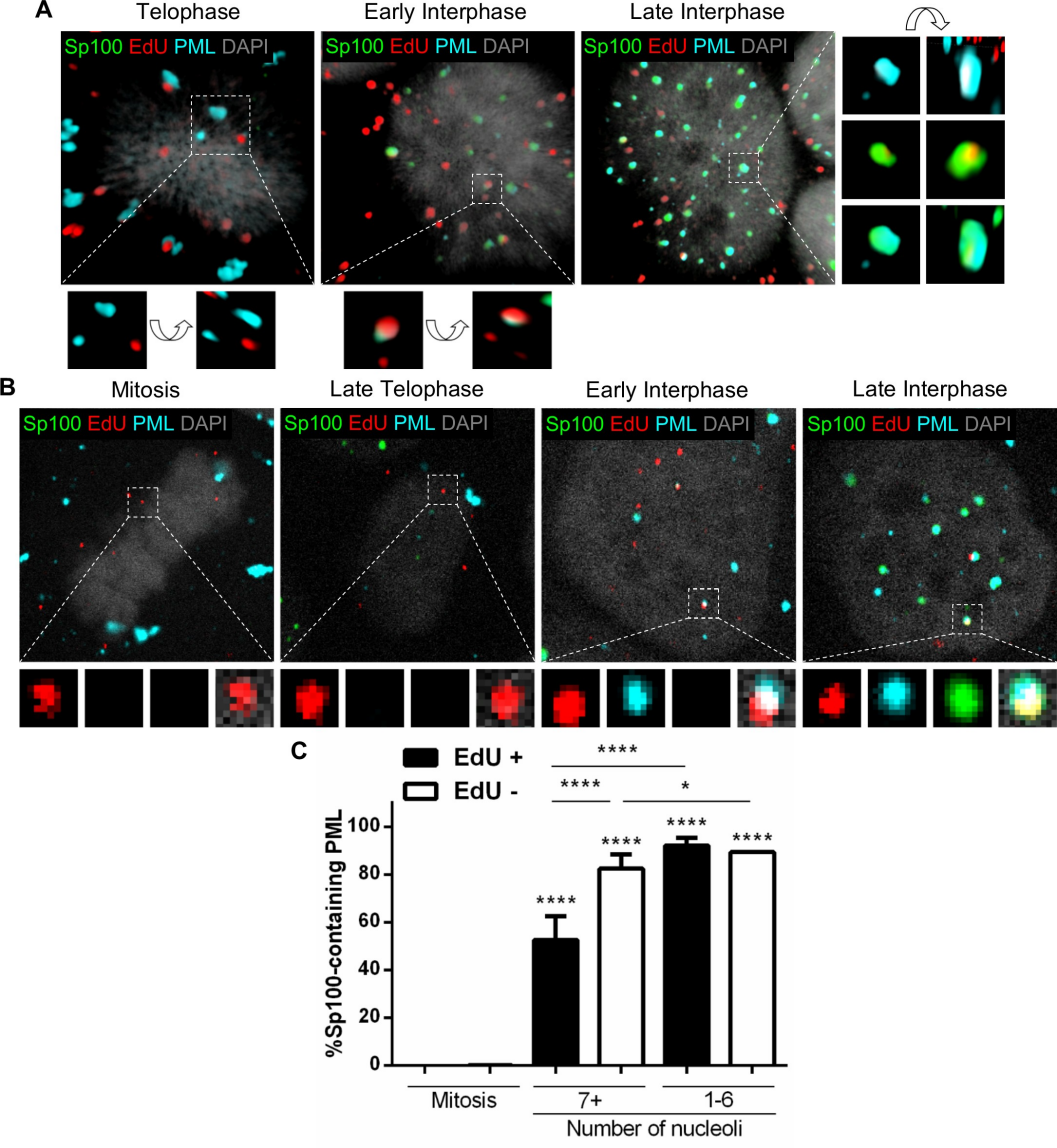
Sp100 recruitment to the viral genome is delayed compared to PML protein. (A and B) HaCaT cells were infected with EdU-labeled PsVs for 24 h, fixed, permeabilized, and treated with Click-iT reaction buffer with AF555 dye to stain EdU-labeled pseudogenomes (red). Next, the cells were incubated with mouse anti-PML protein antibody (cyan) and rabbit anti-Sp100 antibody (green) and mounted in DAPI (white). (A) Images show 3D reconstruction of high resolution z-stacks. Close-up images were rotated at a 45° angle on the x, y, z axes in the 3D-rendered images. (B) Representative confocal images of the following quantification. (C) Percent Sp100-containing PML was determined by manually counting the PML protein puncta co-localizing with Sp100 puncta also co-localizing with EdU puncta (EdU+) or not (EdU-) associated with condensed chromosomes in mitotic cells or nuclear-localized in interphase cells. Nucleoli were also counted: 7+ nucleoli corresponds to early interphase, 1–6 to late interphase. Results are shown as average of 3 independent experiments and SEM, with 30–50 cells of each condition (mitotic, early and late interphase cells) collected in z-stacks spanning the whole nucleus. (C) Mitosis: EdU+ = 0% ± 0%; EdU- = 0.13% ± 0.13%. 7+ nucleoli: EdU+ = 52.56% ± 9.95%; EdU- = 82.48% ± 5.83%. 1–6 nucleoli: EdU+ = 92.10% ± 3.19%; EdU- = 89.38% ± 0.08%. *p* value was determined using Student’s *t*-test. ****: *p* < 0.0001. *: *p* < 0.05. ns: *p* > 0.05.

Another major component of PML NBs is Sp100. While the presence of PML protein is critical for HPV transcription, Sp100 is known to restrict HPV transcription and replication [26,28]. Therefore, we hypothesized that Sp100 is recruited with a delay compared to the recruitment of PML protein after the viral genomes becomes accessible. To address this, we performed immunofluorescent staining on EdU-labeled PsV-infected HaCaT cells to detect EdU-labeled viral pseudogenomes (red), PML protein (cyan), and Sp100 (green) and acquired high resolution images of several z-stacks combined by 3D reconstruction (Fig 7A) and confocal images (Fig 7B). During mitosis, PML protein formed cytosolic aggregates and Sp100 signal was not detected. In early interphase, Sp100 was detectable but was not seen co-localized with EdU puncta co-localizing with PML protein. During late interphase, both PML protein and Sp100 co-localized with EdU puncta, engulfing the signal in a similar manner as observed in Fig 2B. We quantified the number of Sp100-containing PML foci for the presence or absence of EdU puncta in the same infected cells (Fig 7C). In early interphase cells, 53% of EdU puncta co-localized with PML protein foci containing Sp100, while the rest co-localized with PML protein only. In contrast, in late interphase cells, nearly all EdU puncta co-localized with PML protein and Sp100 (92%). Interestingly, EdU-negative PML/Sp100 foci are nearly as abundant in early and late interphase cells (82% and 89%, respectively). It is important to note that EdU puncta were never seen to co-localize with Sp100 alone. Therefore, our data suggest that Sp100 is recruited with a delay to the viral genome and PML protein. More fascinating, this delay seems to be specific for PML NBs forming around incoming viral genome.

**Fig 8:**
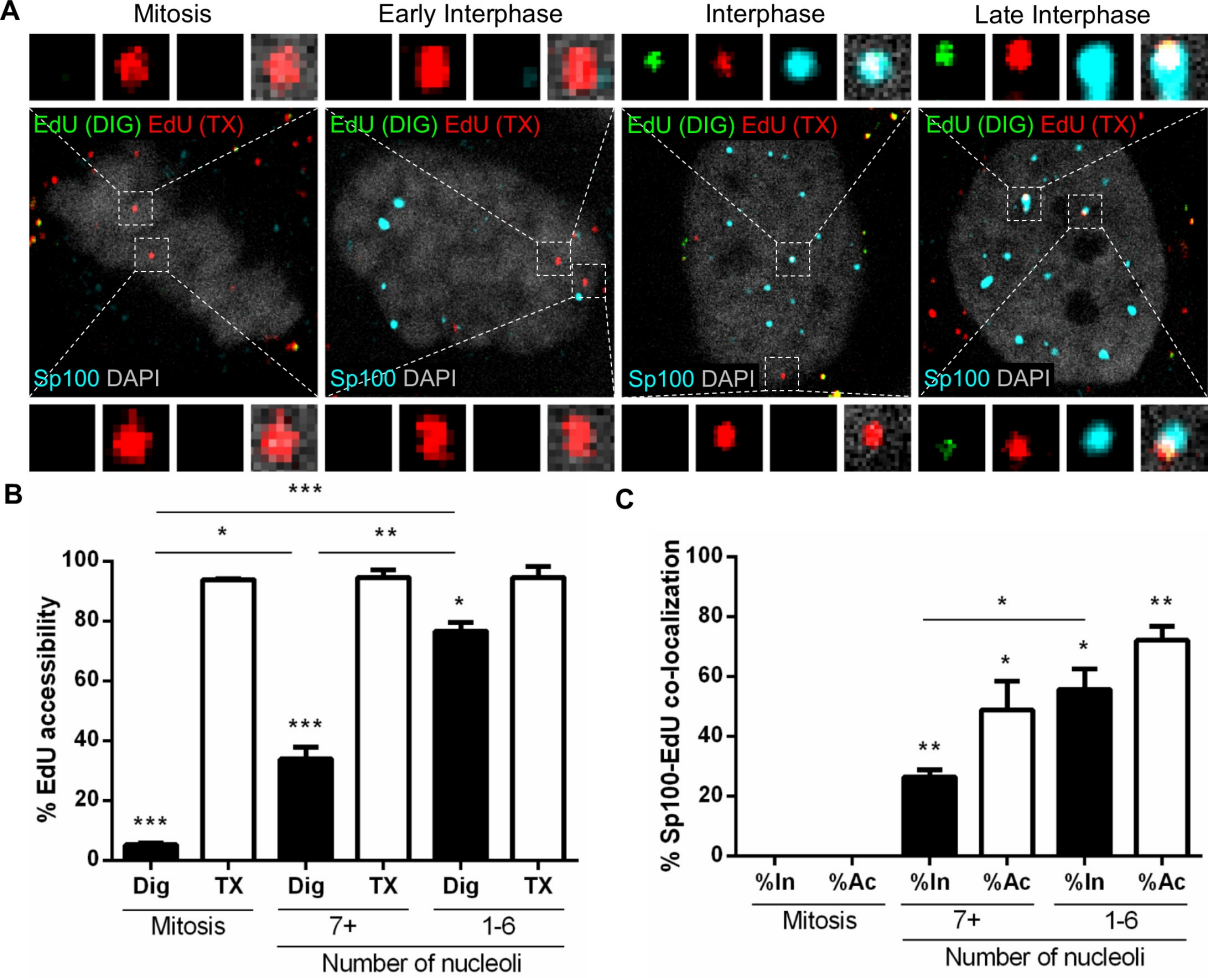
Sp100 is recruited after the viral genome becomes accessible to small molecular dyes. (A) HaCaT cells were infected with EdU-labeled PsVs for 24 h, fixed, permeabilized with 0.625 μg/mL digitonin and treated with AF555 (green) in Click-iT reaction buffer. Next, the cells were permeabilized again with 0.5% TX-100 and treated with AF647 (red) in Click-iT reaction buffer. Lastly, cells were incubated with rabbit anti-Sp100 antibody (cyan) and mounted with DAPI (white). (B and C) Percent accessibility of viral genome (B) was determined by manually counting the number of red only (inaccessible [In]) or red/green (accessible [Ac]) EdU puncta over the total number of EdU puncta associated with condensed chromosomes in mitotic cells or nuclear-localized in interphase cells in digitonin- or TX-100-permeabilized cells. Nucleoli were also counted: 7+ nucleoli corresponds to early interphase, 1–6 to late interphase. Co-localization of Sp100 and EdU (C) was quantified by manually counting the number of EdU puncta that co-localized with Sp100 signal over the total number of accessible or inaccessible EdU puncta associated with condensed chromosomes in mitotic cells or nuclear-localized in interphase in digitonin-permeabilized cells. Results are shown as average of 3 independent experiments and SEM, with 30–50 cells of each condition (mitotic, early and late interphase cells) collected in z-stacks spanning the whole nucleus. (B) Mitosis: Dig: %Ac = 5.22% ± 0.69%; TX: %Ac = 93.87% ± 0.38%. 7+ nucleoli: Dig: %Ac = 33.98% ± 3.89%; TX: %Ac = 94.60% ± 2.57%. 1–6 nucleoli: Dig: %Ac = 76.67% ± 2.86%; TX: %Ac = 94.59% ± 3.64%. (C) Mitosis: %In at Sp100 = 0% ± 0%; %Ac at Sp100 = 0% ± 0%. 7+ nucleoli: %In at Sp100 = 26.49% ± 2.46%; %Ac at Sp100 = 48.95% ± 9.61%. 1–6 nucleoli: %In at Sp100 = 55.79% ± 6.74%; %Ac at Sp100 = 72.22% ± 4.66%. *p* value was determined using Student’s *t*-test comparing Interphase to Mitosis. ****: *p* < 0.0001. ***: *p* < 0.001. **: *p* < 0.01. *: *p* < 0.05. ns: *p* > 0.05.

Next, we examined the recruitment of Sp100 with PML NBs as a function of viral genome accessibility using differential staining to distinguish between inaccessible EdU-labeled pseudogenomes (red), accessible pseudogenomes (red/green), and Sp100 (cyan) within cells undergoing mitosis or during early or late interphase (Fig 8A). EdU puncta showed a very reproducible pattern of accessibility as previously observed (Fig 8B). We quantified the number of inaccessible (In) or accessible (Ac) EdU puncta that co-localized with Sp100 in mitosis, early interphase (7+ nucleoli), and late interphase (1–6 nucleoli) in digitonin-treated cells (Fig 8C). Sp100 signal was not detected in mitotic cells. Only a marginal number of inaccessible EdU co-localized with Sp100 during early interphase (26%), which significantly increases in late interphase (56%). Sp100 co-localized with accessible EdU in early interphase and more in late interphase (49% and 72%, respectively). Taken together, these results suggest that Sp100 is recruited to incoming viral genomes and PML protein after viral genomes become accessible.

### Mutations in SIMs on L2 protein render pseudovirions defective for nuclear delivery

**Fig 9:**
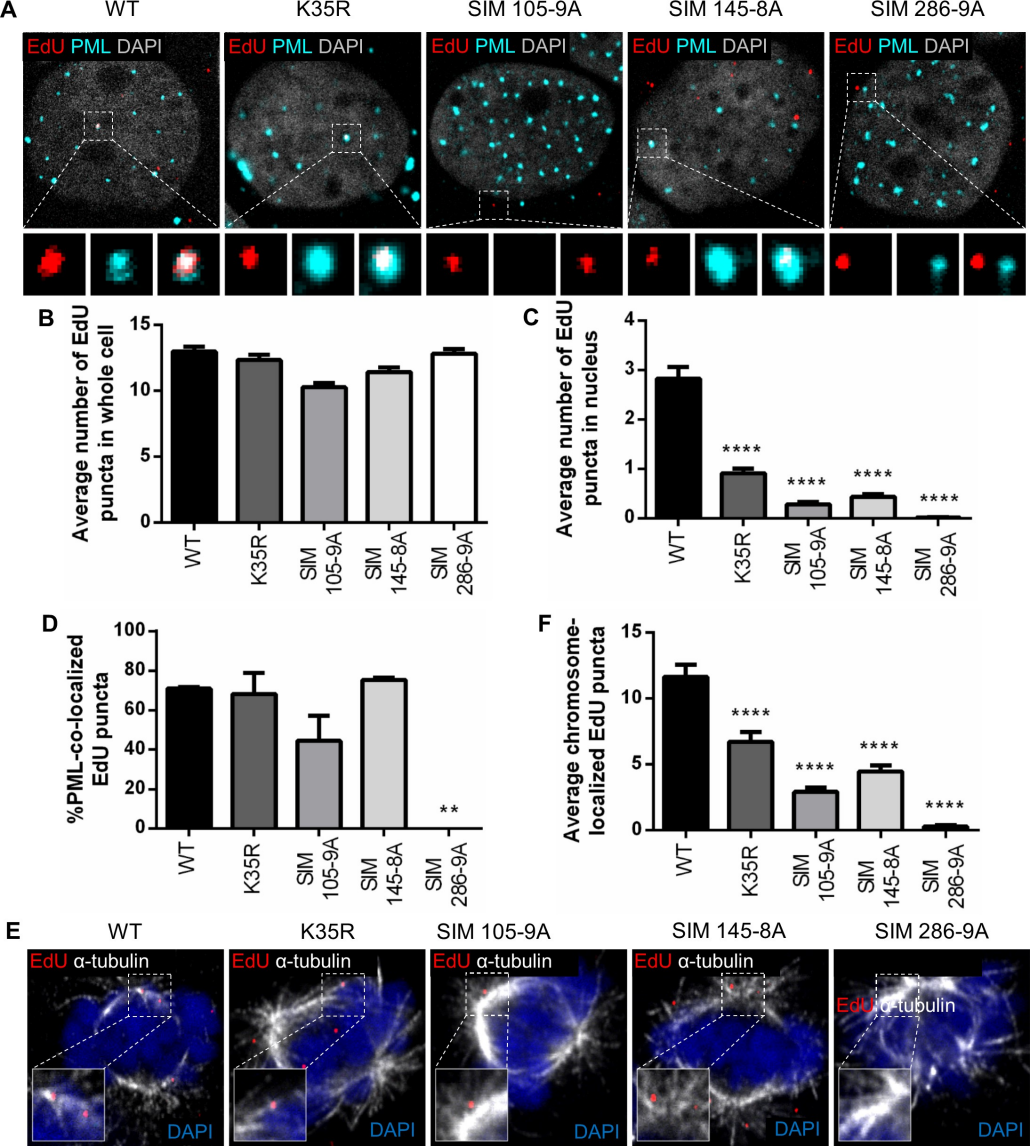
Mutations in SUMO motifs on L2 protein render pseudovirions defective for nuclear delivery. (A and E) HaCaT cells were infected with EdU-labeled WT or mutant PsVs for 24 h, fixed, permeabilized, and treated with AF555 (red) in Click-iT reaction buffer. Next, cells were incubated with rabbit anti-PML protein antibody (cyan) (A) or mouse anti-α-tubulin antibody (white) (E) and mounted with DAPI (white (A), blue (E)). (A) Representative confocal images of EdU and PML localization in interphase cells. (E) Representative confocal images of EdU on microtubules in mitotic cells. (B) Number of EdU in whole cell was determined by manually counting EdU puncta present in z-stacks spanning whole nucleus of interphase cells. WT = 12.99 ± 0.38; K35R = 12.35 ± 0.39; SIM 105-9A = 10.27 ± 0.35; SIM 145-8A = 11.44 ± 0.36; SIM 286 = 9A = 12.83 ± 0.35. (C) Number of EdU in nucleus was determined by manually counting EdU puncta present in z-stacks spanning the whole nucleus of interphase cells. WT = 2.83 ± 0.24; K35R = 0.91 ± 0.10; SIM 105-9A = 0.29 ± 0.43; SIM 145-8A = 0.43 ± 0.06; SIM 286 = 9A = 0.02 ± 0.01. (D) Percent co-localization of EdU and PML protein was determined by manually counting the number of EdU puncta that co-localized with PML protein signal over the total number of EdU puncta present in z-stacks spanning the whole nucleus of interphase cells. WT = 70.93% ± 0.69%; K35R = 68.12% ± 10.58%; SIM 105-9A = 44.48% ± 12.66%; SIM 145-8A = 75.28% ± 1.09%; SIM 286 = 9A = 0% ± 0%. (F) Number of EdU localized on chromosomes was determined by manually counting the number of EdU puncta that co-localized with mitotic chromosomes in z-stacks spanning whole mitotic cells. WT = 11.66 ± 0.92; K35R = 6.73 ± 0.81; SIM 105-9A = 2.92 ± 0.33; SIM 145-8A = 4.47 ± 0.48; SIM 286 = 9A = 0.29 ± 0.10. Each quantification is shown as average of 2 independent experiments and SEM, with 100 cells in each condition and experiment. *p* value was determined using Student’s *t*-test comparing mutants to WT. ****: *p* < 0.0001. **: *p* < 0.01. ns: *p* > 0.05.

Other DNA viruses have been shown to target PML protein via SUMO interaction with viral proteins, such as HSV-1 ICP0 and ADV E1A [15,16]. Considering that HPV L2 protein is interacting with cellular factors during trafficking [32,39], we hypothesized that L2 is also interacting with and recruiting PML protein and possibly Sp100. A SUMO conjugation motif (K35), one highly conserved SIM (aa286-289), and two putative SIMs (aa105-109 and aa145-148) have been identified on L2 protein [24,41]. Therefore, we sought to investigate whether at least one or more of these sites played a role in recruiting PML NB proteins to viral genomes. To test this, we generated EdU-labeled PsVs carrying mutations in L2 protein (S1 Table). We performed site-directed mutagenesis on our L2 expression plasmid to disrupt the SUMO conjugation motif with a residue substitution K35R [41]. The L2 mutants disrupting each SIM (SIM 105-9A, SIM 145-8A, SIM 286-9A) have recently been described [24]. All mutant L2 proteins were efficiently incorporated into PsVs and the mutations did not affect PsV binding to the cell surface (S2 Fig). Next, we infected HaCaT cells with WT or L2 mutant EdU-labeled PsVs and performed immunofluorescent staining to detect PML protein (cyan) and viral pseudogenomes (red) (Fig 9A). Although cells were infected with same amounts of viral genome equivalents (vge) and comparable numbers of EdU puncta were detected in whole cells (Fig 9B), the number of EdU puncta present in the nucleus was dramatically decreased in cells infected with L2 mutant PsVs compared to WT (Fig 9C). Consequently, we observed a significant reduction in the number of L2 mutant EdU puncta co-localized with PML protein compared to WT. However, when we normalize the number of EdU co-localized with PML protein to the total number of EdU puncta in the nucleus of infected cells, there is no significant difference between WT and L2 mutants, apart from SIM 286-9A as almost no EdU puncta were observed in the nucleus (Fig 9D). These data imply that L2 mutant PsVs are not delivered into the nucleus as efficiently as WT PsVs. To test this, we examined the association of viral genomes with mitotic microtubules and chromosomes in HaCaT cells infected with WT and L2 mutant EdU-labeled PsVs by immunofluorescent staining to detect EdU (red) and microtubules (white) (Fig 9E). We observed a significant loss of EdU puncta associated with condensed chromosomes during mitosis in cells infected with L2 mutant PsVs compared to WT PsV-infected cells (Fig 9F). Taken together, these findings suggest that the mutations on L2 protein render the PsVs deficient for nuclear delivery, in a similar phenotype to a previously identified mutant R302/5A [30].

## Discussion

PML protein has been shown to be critical for the retention of HPV genomes in the nucleus of host cells and transcription [25,27]. However, the temporal recruitment of PML NB proteins and how they associate with incoming viral genomes is still poorly defined. The findings described herein suggest that PML protein and SUMO-1 are recruited to and assemble around incoming viral genomes after nuclear delivery and completion of mitosis but prior to the genome becomes accessible in the nucleus. Furthermore, L1 protein accompanies the viral genome into the nucleus followed by PML protein recruitment during early interphase, but L1 protein becomes lost as the cell progresses through interphase, while L2 protein remains with the viral genome. The transcriptional repressor Sp100 showed a delayed recruitment to viral genomes after the viral genome becomes accessible. Lastly, we determined that disruptions in the SIMs of L2 protein result in varying degrees of deficient nuclear delivery of incoming viral genomes.

The recruitment of PML protein towards incoming viral genomes rather than viral genomes targeting preformed PML NBs is a critical aspect for understanding how HPV genomes are delivered to the nucleus. During mitosis, HPV-harboring vesicles do not associate with MAPPs, or only what seems to be incidental and only transient. MAPPs are still predominantly cytosolic when viral genomes are delivered to the nucleus. This was specifically brought to light using high resolution microscopy as only rotating the images in the three-dimensional plane could reveal that EdU puncta do not truly co-localize with PML protein aggregates in mitotic cells, whereas confocal microscopy could not always distinguish large PML protein aggregates from EdU puncta [44]. At the moment, we cannot completely rule out that PML protein below our limit of detection is co-localizing with viral genomes. However, it seems unlikely as PML protein-deficient HaCaT and HeLa cells are fully capable of delivering viral genomes to the nucleus [25,27]. We observed that PML protein then translocates into the nucleus in early interphase and targets viral genomes to form PML NBs. Therefore, unlike what was recently suggested by Broniarczyk *et al*., our findings suggest that PML protein is not involved in nuclear delivery of HPV genomes, but rather starts playing a role in the nucleus, after viral genomes have already been delivered, which confirms other previous findings [25,27,44]. This is further supported by the recent findings by the Boutell group who observed the recruitment of PML protein and other PML NB-residing proteins to HSV-1 incoming genomes after nuclear entry but prior to the initiation of lytic replication [19]. High resolution microscopy also showed the structure of PML protein entrapping HSV-1 genomes similarly to our observation with PML protein engulfing HPV pseudogenomes.

The temporal recruitment and structure of PML protein surrounding viral genomes offer support for our hypothesis that PML protein provides a protective environment for the viral genome against innate and intrinsic immune sensors, rather than an environment favoring transcription as previously suggested [25]. Indeed, our previous findings suggest that incoming PV genomes can be sensed in PML protein-deficient cells and subsequently targeted for degradation [27]. IFI16 (IFN Gamma Inducible Protein 16) has been a major candidate for viral DNA sensing in host cell nucleus and restricts HSV-1 and HPV18 replication and transcription [45]. However, more recently, the repression of HSV-1 replication was shown to occur rapidly after association with PML NBs and independently of IFI16 and induction of ISG (IFN-stimulated gene) expression [19]. However, in the context of HPV infection, knocking down IFI16 did not prevent genome loss in HaCaT cells [27]. Another possible candidate is the Myb-related transcription factor MYPOP that has recently been shown to sense incoming HPV DNA and L2 protein and subsequently inhibit early gene expression [46]. Here, PML protein may compete for binding of such restriction factors to L2 protein to protect the infectious HPV complex and prevent transcriptional repression.

Nevertheless, PML protein seems to protect the viral genome from such a fate. Our previous work demonstrated that the viral genomes are delivered to the nucleus of target cells in a membrane-bound vesicular compartment [30,32]. We have previously shown that the egress from the transport vesicle by an unknown mechanism is slow, resulting in a delay of transcription by four to five hours when compared to a transfection method that requires mitosis for nuclear delivery [30]. The delay strongly suggests that transcriptional activity requires an additional step, which is presumably the egress from the transport vesicles. However, we are aware that a minority (5%) of viral genomes present on mitotic chromosomes is accessible to staining in differentially permeabilized cells and could be the infectious ones. However, we believe that this level of background is most likely due to the extensive processing required for two sequential immunofluorescent stainings. Indeed, in the absence of immunofluorescent processing, viral genome is completely protected from degradation by nucleases when the cells were arrested in mitosis [30]. Furthermore, the work done by the Schelhaas, Campos, DiMaio, and Tsai groups, using a BirA-based system, supports the importance of the transport vesicle for productive infection [47–50]. Therefore, upon nuclear delivery, the viral genome is already presumably protected from DNA sensors within the transport vesicle. The formation of the PML protein structure around the genome-harboring vesicle allows for egress of the viral genome, while remaining protected. We speculate that this step allows for the initiation of transcription responsible for the primary amplification of viral genomes resulting in the establishment of infection [51]. Interestingly, we observed a delayed recruitment of Sp100 to viral genomes when compared to PML protein. This delayed recruitment of Sp100 seems to be specific for HPV-harboring PML NBs and occurs mostly after viral genomes become accessible, although, due to the unknown specificity of our Sp100 antibody, we cannot rule out that low but undetectable levels of Sp100 are present early. It has been demonstrated that Sp100 restricts HPV18 early transcription during establishment of infection [26]. It is attractive to speculate that HPV exploits PML NBs to regulate early transcription, PML protein allowing early transcription to establish infection and delayed Sp100 recruitment helping transition to the maintenance phase. The McBride group also demonstrated that Sp100 does not seem to be involved in the maintenance phase [28]. Additionally, it restricts viral processes in later stages of infection, during the differentiation-induced viral amplification [28]. Our data strongly suggest that L2 protein mediates the recruitment of PML protein to viral genome prior to complete release within the nucleus. It has become clear in recent studies that L2 protein is ultimately lost after orchestrating viral delivery [31]. Therefore, we assume that PML NB association is lost after the next round of mitosis, which would be consistent with the findings by Stepp *et al*. [26,28]. However, further experimentation is needed to test this assumption and to link PML NB composition and transcriptional activity of incoming viral genome.

Our recent work also focused on the role of the capsid proteins during trafficking and nuclear delivery, more specifically the L1 protein. We have shown that L1 protein remains associated with the viral genome in the nucleus of infected cells [31]. We demonstrated that L1 protein directly interacts with the viral DNA and a transmembranous L2 protein while inside a transport vesicle during trafficking and after nuclear delivery. In addition, reactivity of conformationally-dependent antibodies provided evidence that L1 protein was likely arranged as capsomeres while it accompanies the viral genome to the nucleus. Herein, we observed that L1 protein remains associated with the viral genome within the nucleus and that PML protein is recruited and assembles around it. In late interphase, L1 protein dissociates, timed with release of the viral genome, while the viral genome remains at PML NBs. At this time, there is no evidence to suggest that L1 protein plays a role beyond just incidental trafficking. Therefore, we only refer to the loss of L1 protein as a marker for the point in time that coincides with release of the viral genome in the nucleus.

Considering that L1 protein resides within the lumen of the transport vesicle with the viral genome during trafficking into the nucleus, our lab and others have hypothesized that L2 protein is involved in recruiting PML NB proteins. Indeed, L2 protein is already known to contain many domains involved in interacting with cellular factors to facilitate trafficking [32,38,39]. These domains also include SUMO conjugation and interacting motifs [24,41]. SUMOylation is such a critical step in the formation of PML NBs and the recruitment of additional proteins, therefore it is thought that L2 protein may be responsible for recruiting PML protein and Sp100 via SUMO interactions. The Florin group identified three SIMs on L2 protein, at residues 105–9, 145–8, and 286–9. The latter was found to interact with cellular SUMO-1/2 and to be essential for interaction with PML protein as PsVs carrying a disrupted SIM resulted in a decrease in PML co-localization [24]. However, they also noted that infection with the mutant PsVs exhibited reduced amounts of L2 protein and viral genomes in the nucleus, suggesting the mutation may affect events upstream of PML accumulation, such as nuclear localization and retention. The Schelhaas group also investigated the nuclear delivery of the SIM 286–9 and observed that the mutant PsVs were impaired in interacting with mitotic chromosomes, although the SIM 286–9 mutation had been shown to result in clear nuclear accumulation and loss of PML co-localization after L2 overexpression [24,47]. Herein, we show that all of the mutants used in the study were deficient in nuclear delivery. These mutants had a similar pattern to the nuclear retention mutant, 302/5A, that failed to traffic along spindle microtubules, which also resulted in lower infectivity [30]. L2 protein is very compact and harbors many important domains on its C-terminus [23,29,30,32,38,39,47,52]. Therefore, it is a possibility that by mutating the SIMs, we may have disrupted other domains on L2 protein that are essential for cellular trafficking or nuclear delivery. We also cannot exclude defects in proper assembly and early events of the infectious entry process. Therefore, at this time, we are unable to directly test whether the SIMs are responsible for PML recruitment, although these regions are still of particular interest as they all seem to be important for the delivery of the viral genome to the nucleus.

In summary, we present herein a promising model defining the order of events following nuclear delivery of HPV genomes. We demonstrated that PML protein and SUMO-1 are recruited to and assemble around viral genomes that still reside within the transport vesicle in early interphase cells. As L1 protein accompanies the viral genome to the nucleus, it also localizes at PML protein, but is lost in later stages of interphase along with the transport vesicle and release of the viral genome into the nucleus. Then, Sp100 is recruited to viral genomes and also engulfs them in a PML NB structure. Further studies will be necessary to link HPV transcriptional regulation to PML NB composition. However, our study defines these events, thus providing new insights into the kinetics of the primary HPV infection and how HPV relies on the specific temporal recruitment of various factors necessary to promote infection.

## Materials and methods

### Cell lines

The 293TT cells (a kind gift of Dr. John T. Schiller, Laboratory of Cellular Oncology, National Cancer Institute, Bethesda) used for the generation of pseudovirions were cultured in Dulbecco’s Modified Eagle Medium (DMEM) supplemented with 10% fetal bovine serum (FBS), non-essential amino acids, L-Glutamax, and antibiotics. HaCaT cells (purchased from the American Type Culture Collection) used in the infection studies were grown in low glucose DMEM containing 5% FBS and antibiotics.

### Generation of HPV16 pseudovirions

HPV16 pseudoviruses (PsVs) encapsidating a green fluorescent protein (GFP) expression plasmid (pfwB) were generated in 293TT cells as previously described using puf16L1 and puf16L2HA-3’ [53–56]. The pfwB plasmid was a kind gift from Dr. John Schiller (National Cancer Institute, Bethesda, MD). The L1 and L2 expression plasmids harbor codon-optimized genes and were kindly provided by Dr. Martin Müller (Deutsches Krebsfoschungszentrum, Heidelberg, Germany) [57]. For detection of pseudogenomes by IF staining, pseudogenomes were labeled by supplementing the growth media with 100 μM 5-ethylnyl-2’-deoxyuridine (EdU) at 6 hours post transfection during PsV generation, as previously described [58]. Viral DNA within the virions was isolated using NucleoSpin Blood QuickPure (Machery-Nagel; #740569.250) supplemented with 4 μM Ethylenediamineteraacetic acid (EDTA) and Dithiothreitol (DTT) and genome copy number was quantified by quantitative PCR (qPCR). Capsid composition was verified by western blot analysis. For all experiments, 100 to 300 vge/cell were added.

### Generation of mutant PsVs

Point mutation K35R in L2 [41] was generated by site-directed mutagenesis using a pair of complementary PCR primers specific to codon-optimized plasmid puf16L2HA-3’ using the *PfuUltra* II Hotstart DNA Polymerase 2x Master Mix (Stratagene; #600630) following manufacturer’s protocol. The entire plasmid was amplified during the PCR reaction and the PCR products were digested with DpnI to remove methylated template DNA. The PCR products were transformed and the mutation was confirmed by sequencing (Macrogen). L2 expression plasmids harboring SIM mutations (pCDNA16L2-ΔSIM105-109, pCDNA16L2-ΔSIM145-148, pShell16L1L2-ΔSIM286-289) were a kind gift from Dr. Luise Florin (University of Mainz, Germany) [24]. Mutant L2 expression plasmids were used to generate mutant PsVs as described above. Capsid composition and vge were determined by western blot and qPCR as described above. Same amounts of vge were used in experiments comparing WT and mutant PsVs.

### Antibodies and reagents

Antibodies used for the IF studies were as follows: rabbit polyclonal antibody (pAb) anti-PML (BETHYL; #A301-167A), mouse monoclonal antibody (mAb) anti-PML (Santa Cruz Biotechnology; #sc-966), rabbit pAb anti-TGN46 (Thermo Scientific; #PA5-23068), rabbit pAb anti-SUMO-1 (abcam; #ab11672), AlexaFluor (AF) 488-conjugated rabbit pAb anti-GFP (Molecular Probes; #A21311), mouse mAb anti-CD147 (Affinity BioReagents; #MA1-19202), mouse mAb anti-alpha-Tubulin (Cell Signaling; #3873S), AF647-conjugated phalloidin (Molecular Probes; #A22287), and goat AF-labeled secondary antibodies (Life Technologies; #A11034, #A21236). Rabbit polyclonal anti-Sp100 antibody was a kind gift from Dr. Hans Will (Heinrich Pette Institute, Hamburg, Germany) [59]. L1-specific mouse mAb 33L1-7 and rabbit pAb K75 were described previously [60,61]. L2-specific mouse mAb 33L2-1 was also previously described [62]. Click-iT EdU Imaging Kit (Molecular Probes; #C10338) was used for Click-iT reactions to label EdU-labeled pseudogenomes. For western blot analysis, L1 was detected with mouse mAb HPV16 312F and L2 with mouse mAb 33L2-1, combined with peroxidase-conjugated AffiniPure pAb goat secondary anti-mouse (Jackson ImmunoResearch; #115-035-003).

### Immunofluorescent (IF) staining and microscopy

HaCaT cells were grown on coverslips at approximately 50% confluency and infected with EdU-labeled PsVs at approximately 10^6^ viral genome equivalents per coverslips for 24 h at 37°C. Cells were fixed with 4% paraformaldehyde (PFA) for 15 min at room temperature, washed with phosphate-buffered saline (PBS; pH 7.5), permeabilized with 0.5% TX-100 in PBS for 5 min at room temperature, washed with PBS, and blocked with 5% normal goat serum (NGS) for 15 min at room temperature. The Click-iT reaction containing AF555 followed for 30 min at room temperature and protected from light to specifically detect EdU-labeled pseudogenomes [58]. After cells were washed with PBS, they were incubated with primary antibodies in 2.5% NGS for 30 min at 37°C in a humidified chamber. After extensive washing with PBS, the cells were incubated with AF-labeled secondary antibodies in 2.5% NGS for 30 min at 37°C in a humidified chamber. After another round of extensive washing with PBS, the cells were mounted in ProLong Gold antifade reagent with DAPI (4’, 6’-diamidino-2-phenyllindole; Invitrogen; #P36931). Confocal images were acquired in single-slices or z-stacks with Nikon A1R confocal microscope using a 100X objective and NIS Elements software. Number of EdU-labeled pseudogenomes in nuclei was quantified in z-stacks spanning the whole nucleus. Results are expressed in average number of EdU or percent of EdU-labeled viral pseudogenome in the nucleus ± standard error of the mean (SEM). High resolution images were acquired with Nikon N-SIM E Super Resolution microscope using 100X objective. Several z-stacks spanning the whole nucleus were acquired and assembled using 3D reconstruction in NIS Elements software. All images from individual experiments were acquired under the same laser power settings and enhanced uniformly in Adobe Photoshop.

### Immunofluorescent (IF) microscopy after selective permeabilization

HaCaT cells were grown on coverslips to 50% confluency and infected with EdU-labeled PsVs for 24 h at 37°C. Cells were fixed with 4% PFA for 15 min at room temperature, washed with PBS, and selectively permeabilized with 0.625 μ/mL of digitonin in water for 5 min at room temperature, washed with PBS, and blocked with 5% NGS for 15 min at room temperature. Cells were treated with the first Click-iT reaction with AF555 for 30 min at room temperature protected from light. Cells were washed with PBS, permeabilized with 0.5% TX-100 for 5 min at room temperature, and blocked with 5% NGS for 15 min at room temperature. Cells were treated with the second Click-iT reaction with AF647 for 30 min at room temperature protected from light. Cells were washed with PBS and incubated with primary and secondary antibodies to detect protein of interest as described above. Cells were extensively washed and mounted with DAPI. Differential staining of EdU-labeled pseudogenome using sequential Click-iT reactions in selectively permeabilized HaCaT cells was previously described in greater details [30]. A control experiment was performed by treating the cells with 0.5% TX-100 in both permeabilization steps. In a parallel experiment, cells were incubated with primary Ab anti-TGN46 in 2.5% NGS for 30 min at 37°C in a humidified chamber following the first Click-iT reaction. Cells were washed extensively and incubated with secondary AF488-labeled Ab in 2.5% NGS for 30 min at 37°C. This parallel experiment acts as a permeabilization control, as previously described [32]. All images were acquired in z-stacks spanning the whole nucleus with Nikon A1R confocal microscope using a 100X objective and NIS Elements software. All images from individual experiments were acquired under the same laser power settings and enhanced uniformly in Adobe Photoshop. Co-localization of EdU puncta with protein of interest was quantified by counting the number of nuclear EdU puncta as a function of single or double EdU staining and co-localization with protein of interest and expressed as percent co-localization of total nuclear accessible or inaccessible EdU puncta ± SEM.

### Cell binding assay

HaCaT cells were grown on coverslips to 70% confluency and equal number of PsVs were allowed to bind to the cell surface for 1 h at 37°C. Cells were stained as described above without the Click-iT reaction. Instead, conformational L1 protein was detected with K75 Ab. Assay was quantified as pixel sum ratio of L1-specific signal on the cell surface to region of interest (ROI) area and expressed as percent of WT (100%) ± SEM. All images were acquired in single slice through the cell with Nikon A1R confocal microscope using a 100X objective and NIS Elements software. All images from individual experiments were acquired under the same laser power settings and enhanced uniformly in Adobe Photoshop.

## Supporting information

S1 Fig Using selective permeabilization and Click-iT reaction chemistry to differentially stain the viral genome with small molecular dyes. HaCaT cells were infected with EdU-labeled PsVs for 24 h, fixed, permeabilized with 0.625 μg/mL digitonin or 0.5% TX-100, and treated with AF555 (green) in Click-iT reaction buffer. Next, the cells were permeabilized again with 0.5% TX-100 and treated AF647 (red) in Click-iT reaction buffer. Lastly, cells were incubated with rabbit anti-TGN46 (cyan) (A) or rabbit anti-PML protein antibody (cyan) (B) and mounted with DAPI.
(TIF)

S2 Fig Mutations in SUMO motifs on L2 protein do not affect binding of pseudovirions. (A) WT and mutant PsVs were denatured and L1 (55 kDa) and L2 (75 kDa) proteins were detected with mouse 312F and 33L2-1 antibodies, respectively, by western blot analysis. (B and C) HaCaT cells were infected with WT or mutant PsVs for 1 h, fixed, permeabilized, and incubated with mouse anti-CD147 antibody (blue) as a membrane marker and rabbit K75 antibody (red) for the specific detection of conformational L1 protein and mounted with DAPI (white). (B) Representative confocal images of the binding assay. (C) Percent binding was determined as pixel sum ratio of L1 signal on the cell surface to ROI area and normalized to WT. Results are shown as average of 2 independent experiments and SEM, with 50 cells in each condition and experiment: WT = 100.00% ± 3.05%; K35R = 68.92% ± 1.27%; SIM 105-9A = 98.57% ± 1.75%; SIM 145-8A = 74.86% ± 7.68%; SIM 286 = 9A = 107.24% ± 12.08%. Statistics were calculated using Student’s *t*-test comparing each mutant to WT and no significant differences were found: ns: *p* > 0.05.
(TIF)

S1 Table Summary of mutations on L2 proteins. WT column shows amino acid sequence of the domain on L2 protein. Mutation column shows the mutated amino acid sequence used for the mutant PsVs.
(TIF)
