# Supplementary figures and images for: PML nuclear body-residing proteins sequentially associate with HPV genome after infectious nuclear delivery
Source: PLoS Pathog. 2019 Feb 25;15(2):e1007590. doi: 10.1371/journal.ppat.1007590 (PMC6405170; doi:10.1371/journal.ppat.1007590)

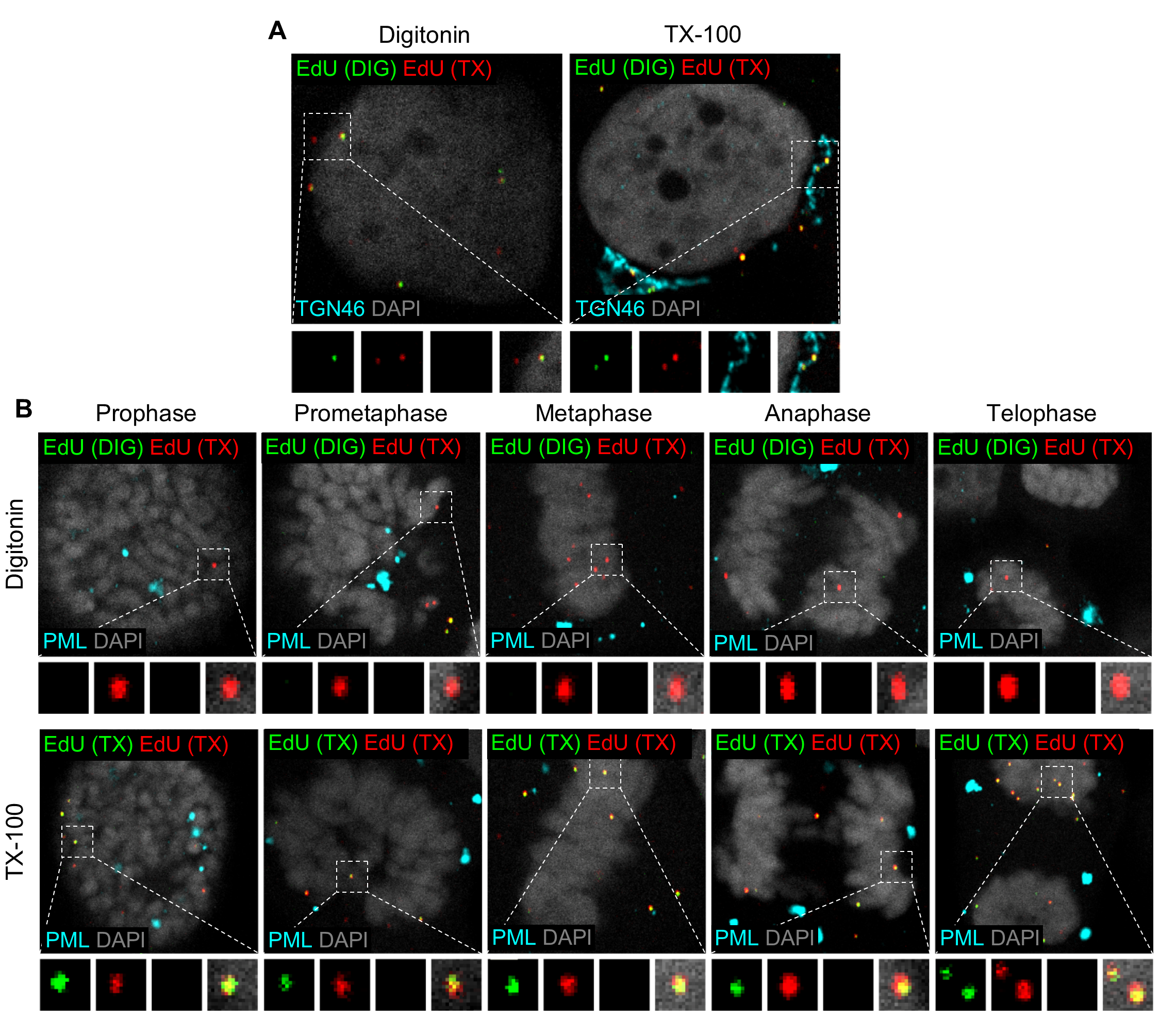

Supplement: S1 Fig — HaCaT cells were infected with EdU-labeled PsVs for 24 h, fixed, permeabilized with 0.625 μg/mL digitonin or 0.5% TX-100, and treated with AF555 (green) in Click-iT reaction buffer. Next, the cells were permeabilized again with 0.5% TX-100 and treated AF647 (red) in Click-iT reaction buffer. Lastly, cells were incubated with rabbit anti-TGN46 (cyan) (A) or rabbit anti-PML protein antibody (cyan) (B) and mounted with DAPI. (TIF) [file ppat.1007590.s001.tif]

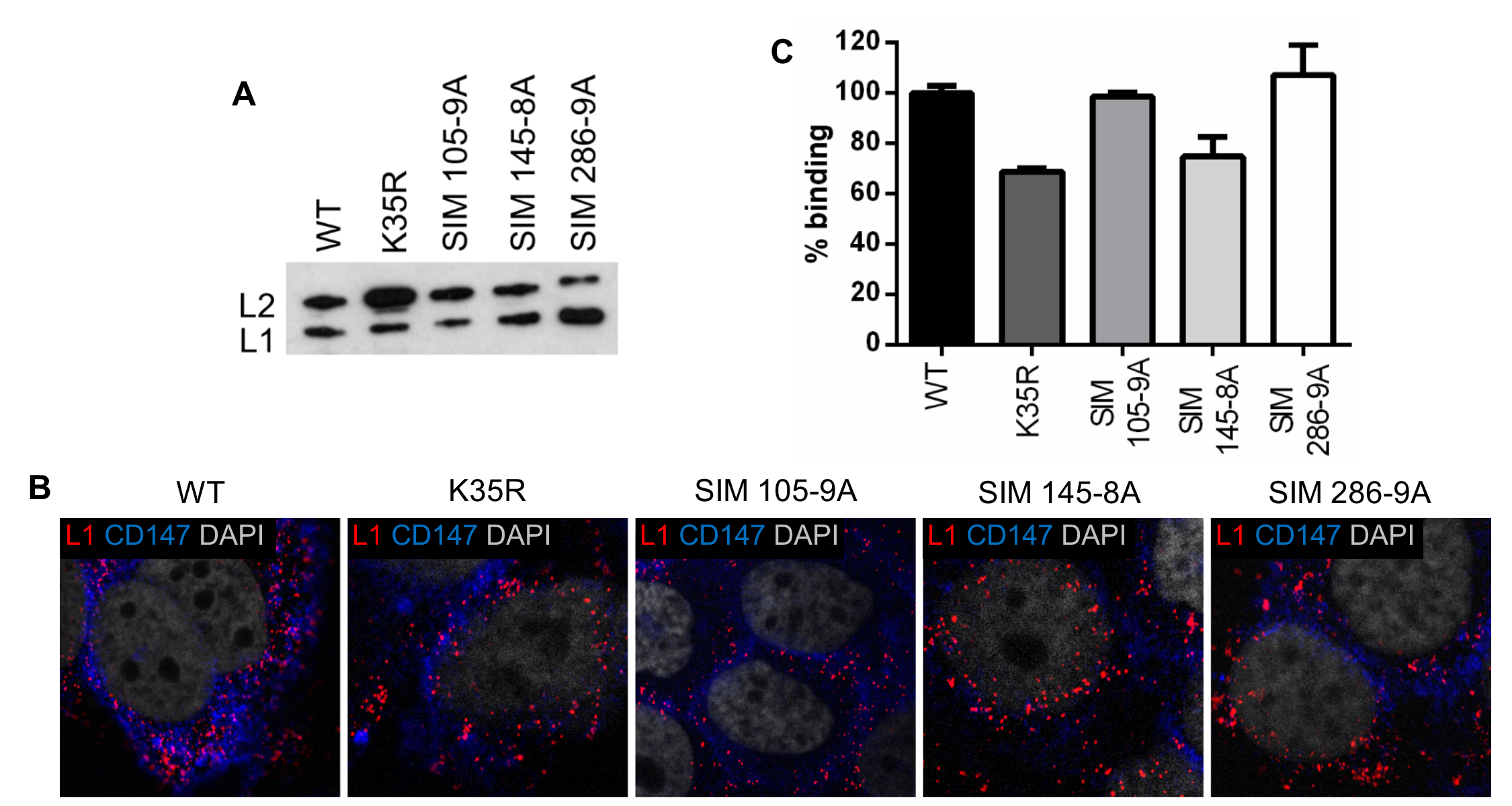

Supplement: S2 Fig — (A) WT and mutant PsVs were denatured and L1 (55 kDa) and L2 (75 kDa) proteins were detected with mouse 312F and 33L2-1 antibodies, respectively, by western blot analysis. (B and C) HaCaT cells were infected with WT or mutant PsVs for 1 h, fixed, permeabilized, and incubated with mouse anti-CD147 antibody (blue) as a membrane marker and rabbit K75 antibody (red) for the specific detection of conformational L1 protein and mounted with DAPI (white). (B) Representative confocal images of the binding assay. (C) Percent binding was determined as pixel sum ratio of L1 signal on the cell surface to ROI area and normalized to WT. Results are shown as average of 2 independent experiments and SEM, with 50 cells in each condition and experiment: WT = 100.00% ± 3.05%; K35R = 68.92% ± 1.27%; SIM 105-9A = 98.57% ± 1.75%; SIM 145-8A = 74.86% ± 7.68%; SIM 286 = 9A = 107.24% ± 12.08%. Statistics were calculated using Student’s t-test comparing each mutant to WT and no significant differences were found: ns: p > 0.05. (TIF) [file ppat.1007590.s002.tif]

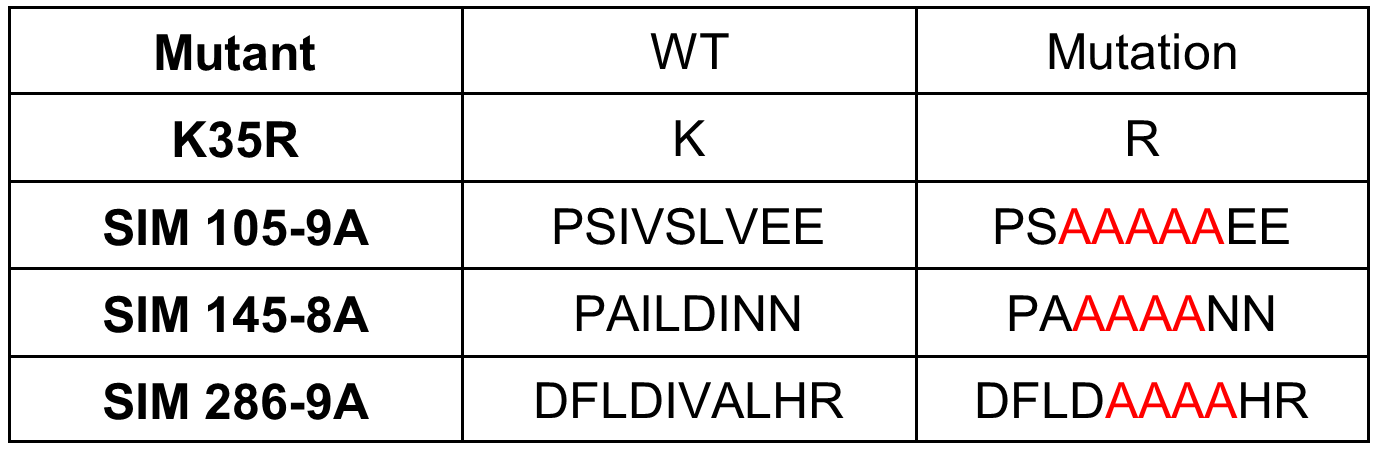

Supplement: S1 Table — WT column shows amino acid sequence of the domain on L2 protein. Mutation column shows the mutated amino acid sequence used for the mutant PsVs. (TIF) [file ppat.1007590.s003.tif]
